# Molecular mechanisms of radioactive iodine refractoriness in differentiated thyroid cancer: Impaired sodium iodide symporter (NIS) expression owing to altered signaling pathway activity and intracellular localization of NIS

**DOI:** 10.7150/thno.57689

**Published:** 2021-04-15

**Authors:** Ji Min Oh, Byeong-Cheol Ahn

**Affiliations:** 1 Department of Nuclear Medicine, School of Medicine, Kyungpook National University, Daegu, Republic of Korea.; 2 Department of Nuclear Medicine, Kyungpook National University Hospital, Daegu, Republic of Korea.; 3 BK21 FOUR KNU Convergence Educational Program of Biomedical Sciences for Creative Future Talents, Department of Biomedical Science, School of Medicine, Kyungpook National University, Daegu, Republic of Korea.

**Keywords:** radioactive iodine refractory thyroid cancer, redifferentiation, signaling pathways, sodium iodide symporter, membrane targeting

## Abstract

The advanced, metastatic differentiated thyroid cancers (DTCs) have a poor prognosis mainly owing to radioactive iodine (RAI) refractoriness caused by decreased expression of sodium iodide symporter (NIS), diminished targeting of NIS to the cell membrane, or both, thereby decreasing the efficacy of RAI therapy. Genetic aberrations (such as *BRAF*, *RAS*, and RET/PTC rearrangements) have been reported to be prominently responsible for the onset, progression, and dedifferentiation of DTCs, mainly through the activation of mitogen-activated protein kinase (MAPK) and phosphoinositide 3-kinase (PI3K)/AKT signaling pathways. Eventually, these alterations result in a lack of NIS and disabling of RAI uptake, leading to the development of resistance to RAI therapy. Over the past decade, promising approaches with various targets have been reported to restore NIS expression and RAI uptake in preclinical studies. In this review, we summarized comprehensive molecular mechanisms underlying the dedifferentiation in RAI-refractory DTCs and reviews strategies for restoring RAI avidity by tackling the mechanisms.

## Introduction

Differentiated thyroid cancers (DTCs), including papillary thyroid cancer (PTC), follicular thyroid cancer (FTC), and Hűrthle cell cancer, arise from thyroid follicular cells and represent >90% of all types of thyroid cancer 1. The development of PTC, accounting for >80% of all thyroid cancers, is majorly caused by *BRAF* (60%), *RAS* (15%), and RET/PTC rearrangements (12%), with these mutations being mutually exclusive, followed by refractoriness to radioactive iodine (RAI) owing to activated mitogen-activated protein kinase (MAPK) signaling pathway 2. FTC, which represents 2%-5% of thyroid cancers, is associated with mostly mutually exclusive *RAS* mutation and PAX8-peroxisome proliferator-activated receptor ɣ (PPARɣ) fusion oncogene; however, Hűrthle cell cancer is genetically different from FTC 2, 3. Although DTCs have a quite favorable prognosis via common therapeutic approaches such as thyroidectomy, RAI therapy, and thyroid-stimulating hormone (TSH) suppressive therapy, local recurrence and distant metastasis inevitably occur up to 20% and 10%, respectively 4. Two-thirds of these patients exhibit loss of iodine-131 (^131^I) uptake initially or gradually, indicating dedifferentiation of the DTC known as RAI-refractory DTC 4, 5.

The 2015 ATA guideline suggested the following criteria as definition of RAI-refractory DTCs 6: 1) patients with malignant/metastatic disease do not concentrate RAI at the time of the initial treatment; 2) patients whose tumors lose the ability to concentrate RAI after previous evidence of RAI-avid disease; 3) patients with RAI uptake concentrated in only some lesions; and 4) patients with metastatic disease that progresses despite substantial concentration of RAI. However, there were some differences regarding details of the definition among researchers owing to reasons such as the number of previous RAI administration, the cumulative dose of RAI, fluorodeoxyglucose (FDG) uptake of the lesion 7-10. These trivial differences in the definition of RAI-refractory DTCs originate from the clinical perspective and can be further refined by clinical experience in the future 4, 11. Cellular dedifferentiation in thyroid cancers eventually causes tumor progression through aggressiveness, metastasis, and reduced RAI uptake with no response to RAI therapy 12. Consequentially, the 10-year survival rate for patients with DTC exhibiting RAI refractoriness is <10% 5.

An important element of RAI therapy is the ability of thyroid follicle cells to take up and concentrate iodide 13, 14. This treatment exploits the unique function of the iodide-metabolizing machinery of thyroid follicular cells as well as the expression of thyroid-specific genes, such as sodium iodide symporter (NIS), thyroglobulin (Tg), TSH receptor (TSHR), and thyroperoxidase (TPO) in addition to thyroid-specific transcription factors such as paired box gene-8 (PAX-8) and thyroid transcription factor-1 (TTF-1) 15, 16. However, the underlying molecular basis of the loss of RAI avidity in RAI-refractory thyroid cancer is the aberrant silencing of expression of both thyroid-specific genes and transcription factor 16. Among these factors, decreased expression of NIS, diminished membrane targeting of NIS, or both, which are mainly caused by genetic and epigenetic alterations and dysregulated signaling pathways, are the major mechanisms underlying RAI refractoriness 1, 17, 18. Genetic alterations in MAPK and phosphoinositide 3-kinase (PI3K)/AKT signaling pathways by point mutations or chromosomal rearrangements are fundamental drivers of the pathogenesis of thyroid cancers and RAI refractoriness 12. In addition to the signaling pathways, epigenetic and genetic alterations of other pathways, including Wnt/ß-catenin and TGF-ß/Smad signaling pathways, are also related to the silencing of expression of thyroid-specific genes, resulting in the failure of RAI therapy 1, 16, 19.

This review comprehensively discusses the molecular mechanisms underlying RAI refractoriness in DTC and strategies for restoring RAI avidity.

### Physiological NIS regulation

NIS expression can be regulated at both transcriptional and posttranslational levels 4. Once TSH binds to TSHR, adenylyl cyclase is stimulated via the Gs-protein, with resulting increase in the levels of cyclic adenosine monophosphate (cAMP). Activated cAMP stimulates both protein kinase A (PKA)-dependent and PKA-independent signaling pathways 13, 20. Once cAMP is activated, PKA increases and phosphorylates the cAMP-responsive element modulator, which is followed by an increase in NIS upstream enhancer (NUE) activity through the PKA-dependent signaling pathway 21. Redox effector factor-1 (Ref-1) is activated through the PKA-independent signaling pathway, and then PAX-8 is stimulated to bind to NUE, resulting in NUE activation 12. TSH-independent mechanisms also alter NIS expression by affecting the binding of PAX-8 to NUE. There are three primary signaling pathways that have been identified. First, activation of transforming growth factor-ß (TGF-ß) stimulates SMAD3, which inhibits the binding of PAX-8 to NUE 22, 23. Second, toll-like receptor 4 (TLR4) stimulates nuclear factor kappa-light-chain-enhancer of activated B cells (NF-κB) p65 which activates PAX-8 to bind NUE 24, 25. Third, pituitary tumor-transforming gene 1 (PTTG1)-binding factor (PBF) interrupts the interaction between PAX-8 and NUE which suppresses NIS expression 26, 27.

Another crucial factor determining NIS functionality is the translocation of NIS from the cytoplasm to the plasma membrane by posttranslational modification 13. Kogai et al. demonstrated that rat thyroid cells stimulated by TSH could induce NIS expression by more than 6-fold at 24 h compared with basal levels 28. However, iodide uptake reached a maximum (>27-fold increase compared with basal levels) at 72 h, showing a time lag between NIS expression and iodide uptake owing to the posttranslational regulation of NIS by TSH stimulation. Additionally, TSH stimulation in rat thyroid cells revealed the localization of NIS on the cell membrane, whereas NIS is localized in the cytoplasm after the removal of TSH stimulation. Therefore, the presence of NIS in the cytoplasm is closely associated with posttranslational regulation, resulting in the reduction of iodide uptake.

### Role of iodide metabolism in physiology

Uptake and concentration of iodide and synthesis of thyroid hormone are the main roles and functions of thyroid follicular cells 29. The expression of thyroid-specific genes, such as *NIS*, *TSHR*, *TPO*, and *Tg*, work together to accumulate and retain iodide in the thyroid follicular cells 16. NIS is localized in the basolateral membrane and mediates the active transport of iodide from the blood stream to the thyroid follicular cells 30. TSHR plays a fundamental role in the regulation of thyroid cell proliferation, differentiation, and targeting for iodide uptake through TSH stimulation 31. TSHR expression is impaired in thyroid cancer tissues, although to a lesser extent than other thyroid-specific genes 32. TPO is a key thyroid enzyme for incorporation of iodide into Tg during thyroid hormone synthesis 16. The decrease in TPO expression in thyroid cancer is related to the rapid loss of iodide caused by the cancer which results in the failure of RAI therapy 33, 34. Tg is the iodoprotein precursor of thyroid hormone in thyroid follicular cells and is considered the best marker of thyroid cancer 35. Thyroid-specific transcription factors such as PAX-8, TTF-1, and thyroid transcription factor-2 (TTF-2) play a crucial role in the regulation of gene transcription activity in thyroid follicular cells 16. Several studies noted different expression levels of thyroid-specific genes and thyroid-specific transcription factors within human thyroid tissue 32, 36, 37. Mostly, the expression of both thyroid-specific genes and thyroid-specific transcription factors decreased in dedifferentiated thyroid cancers. Therefore, the process of restoring RAI avidity to the thyroid cannot be achieved with NIS alone, and additional factors such as thyroid-specific genes and thyroid-specific transcription factors are also required.

Gene transfer with thyroid-specific genes or thyroid-specific transcription factors was used to convert dedifferentiation to redifferentiation in thyroid cancers cancers 38, 39. Mu et al. demonstrated the possibility of gene transfer in thyroid cancers to provide effective RAI therapy 38. The results indicated that cotransfer of *TTF-1* and *PAX-8* genes resulted in the induction of NIS, TPO, and Tg expression followed by RAI accumulation and retention. Another group showed that transfer of the TSHR gene into dedifferentiated thyroid cancer reinduced the expression of thyroid-specific genes followed by RAI uptake 39.

## Major signaling pathways

### MAPK signaling pathway

**Figure 1:**
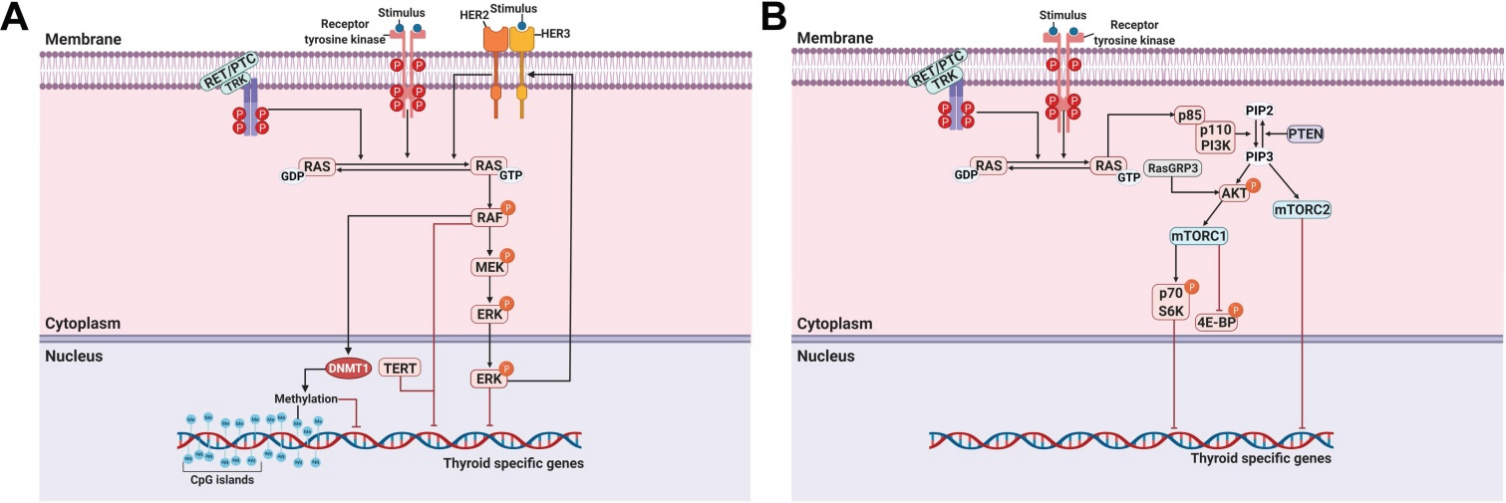
** Principal mechanisms underlying the pathogenesis of thyroid cancer and radioactive iodine (RAI)-refractory in differentiated thyroid cancer.** (A) Mitogen-activated protein kinase (MAPK) signaling pathway. (B) Phosphoinositide 3-kinase (PI3K)/AKT signaling pathway. Abbreviations: 4E-BP: 4E-binding protein; DNMT1: DNA methyltransferase 1; mTORC1: mTOR complex 1; mTORC2: mTOR complex 2; P: phosphorylation; PTEN: phosphatase and tensin homolog; TERT: telomerase reverse transcriptase. Images were created with BioRender.com.

A MAPK can be classified into three main families: extracellular-signal-regulated kinases (ERKs), Jun amino-terminal kinases (JNKs), and p38/stress-activated protein kinases (SAPKs) 40. The MAPK pathway plays a critical role in signaling cascades and transmits its extracellular signal to intracellular targets 40. In thyroid cancer, the MAPK signaling pathway regulates cell proliferation, growth, survival, and dedifferentiation 41. Approximately 70%-80% of human PTCs are connected via mutations of *BRAF*, *RAS*, RET/PTC rearrangement, or *TRK* 42-44. Constitutive activation of the MAPK signaling pathway occurs through the oncoproteins encoded by these genes, which leads to the progression of thyroid cancer (Figure 1A).

Among these mutations, mutation of* BRAF*, which is a highly prevalent oncogene, occurs in approximately 45% (range, 27%-87%) of PTCs and is critical for the aberrant activation of the MAPK signaling pathway 4, 45, 46. In 2005, the first comprehensive multicenter study reported the association of the *BRAF* mutation with poor clinicopathological characteristics of PTC, such as extrathyroidal extension, lymph node metastasis, advanced tumor stage, tumor recurrence, RAI non-avidity, and treatment failure with RAI 47. Subsequently, numerous studies also reported the association between the *BRAF* mutation and loss of RAI avidity with failure of RAI treatment in PTC 48-51. The *BRAF* mutation is highly prevalent in recurrent or metastatic RAI-refractory PTC compared with primary PTC 47, 48, 52, 53. Xing et al. noted that additional surgery or RAI therapy cured patients with recurrent PTC of wild-type *BRAF* 47. However, recurrent PTC patients with the *BRAF* mutation had active disease despite repeated surgery or RAI therapy. Several studies have reported that *BRAF* expression in thyroid cells is closely associated with the impairment of thyroid-specific genes, including NIS; however, this impairment was not totally dependent on the activation of the MAPK signaling pathway 49, 54-56. Liu et al. reported restoring thyroid-specific gene expression by inhibiting the MAPK signaling pathway using U0126 as a MEK inhibitor. This allowed effective RAI therapy against PTC harboring the *BRAF* mutation (Table 1) 56. The results of treatment using BRAF inhibitors demonstrated restoration of thyroid-specific gene expression and RAI uptake in *BRAF*-mutated thyroid cancer cells *in vitro* 19, 57*.* The *in vitro* study was expanded to an *in vivo* study, and it reported that suppression of the MAPK signaling pathway using BRAF and MEK inhibitors in transgenic mice with conditional *BRAF* activation also restored thyroid-specific gene expression and RAI uptake (Table 1) 58. These results have potentially significant clinical ramifications and therapeutic possibilities for patients with RAI-refractory thyroid cancer.

Although administration of BRAF inhibitors such as vemurafenib has shown promising results for RAI-refractory thyroid cancers, the occurrence of adverse effects and primary and/or acquired resistance often limit its applicability for targeted therapy 59. The primary causes of resistance to BRAF inhibitors are mutational events or activation of alternative signaling pathways that reactivate ERK signaling 59. Ofir et al. reported the case of a patient with PTC having RAI-refractory metastatic disease who was treated with vemurafenib for 43 months. Initially, there was a partial response but the disease progressed again. Genetic analysis following the occurrence of drug resistance revealed a new mutation in *NRAS^Q61K^*^60^. Montero-Conde et al. reported that thyroid cancer with the *BRAF* mutation overexpressed human epidermal growth factor receptor 3 (HER3) via autocrine-secreted NRG1 secretion when treated with BRAF inhibitor. This phenomenon triggered the reactivation of both ERK and AKT in thyroid cancer 61. Therefore, overcoming resistance to BRAF inhibitors is crucial for improving therapeutic responses. Cheng et al. applied this strategy for redifferentiation of PTC harboring the *BRAF* mutation and found that the HER inhibitor (lapatinib) prevented the reactivation of the MAPK signaling pathway and sensitized the BRAF/MEK inhibitor. They found that by treating with the HER inhibitor, PTC harboring the *BRAF* mutation was redifferentiated and the expression of thyroid-specific proteins and RAI uptake and therapy were restored (Table 2) 57. Song et al. demonstrated that the effect of vemurafenib treatment was transient and followed by reactivation of the phosphorylation of ERK; however, a combination strategy with a MEK inhibitor overcame the limitations of a transient vemurafenib response in *BRAF*-mutated thyroid cancers 62. Based on a previous study, the same group demonstrated synergistic effects following combination treatment with BRAF inhibitor and MEK inhibitor to increase NIS expression and restore RAI uptake by inhibiting reactivated phosphorylation of ERK in *BRAF*-mutated PTC cells 63.

It is well-known that TSH stimulation has the ability to enhance NIS expression and RAI uptake. Increased levels of TSH are necessary before starting RAI therapy, which can be accomplished either by administering human recombinant TSH or withholding thyroid hormone supplementation. Kleiman et al. reported that *BRAF* silencing of *BRAF*-mutated thyroid cancer could restore thyroid-specific gene expression and RAI uptake with only TSH supplementation 55. In addition, TSH stimulation, along with combination treatment with BRAF inhibitor and histone deacetylase (HDAC) inhibitor, maximized the synergistic effects of restoring thyroid-specific gene expression and RAI uptake in *BRAF*-mutated thyroid cancer 19.

TSH stimulates NIS expression through the accumulation of cAMP, which is a positive regulator of differentiation and proliferation of thyroid cells 64. cAMP can activate the RAC1-MKK3/6-p38ɑ-CHOP signaling pathway for the induction of NIS expression in thyroid follicular cells 64-66. Among these downstream factors, RAC1, known as a small GTP-binding protein, plays a role as a molecular switch in the proliferation, differentiation, and migration of cells 67. Moreover, Faria et al. demonstrated the upregulation of NIS expression via overexpression of the constitutively active mutant *G12V-RAC1* in PTC cells, suggesting that RAC1 activation upregulates NIS expression without TSH stimulation 68. A RAC1b, an alternative splicing variant of RAC1, has been shown to be overexpressed in multiple cancers, suggesting its oncogenic role 69. Several studies reported that the overexpression of RAC1b is closely associated with poor clinical outcomes in PTC and FTC 68, 70, 71. In addition, a possible interaction between RAC1b and *BRAF* mutations may contribute to the downregulation of NIS expression as well as poor clinical outcomes in PTC 68, 71. A possible interplay between RAC1b and *BRAF* mutations may contribute to the downregulation of NIS expression as well as poor clinical outcomes in PTC.

Telomerase reverse transcriptase (TERT), a catalytic protein subunit of telomerase, is a major oncogene in multiple cancers. The presence of *TERT* promoter mutations is found in thyroid cancers, except in medullary thyroid cancer (MTC), suggesting its role in thyroid tumorigenesis and poor clinical outcomes such as recurrence and mortality 72. In addition, the frequency of *TERT* promoter mutations is associated with aggressive characteristics of thyroid cancers. The prevalence of *TERT* promoter mutations is higher in poorly differentiated thyroid cancer (PDTC) and anaplastic thyroid cancer (ATC) than in PTC and FTC 72, 73. Several reports noted that concomitant *BRAF* and *TERT* mutations were closely associated with poor clinical outcomes in PTCs 74, 75. Recently, Liu et al. investigated the genotype patterns of *BRAF* and *TERT* promoter mutations related to the loss of RAI avidity in recurrent PTC 76. They found that the *BRAF* mutation and the concomitant *BRAF* and *TERT* promoter mutations were closely associated with the loss of RAI avidity and impaired the expression of thyroid-specific genes in recurrent PTC (Figure 1A).

Fuziwara et al. reported on the role of FAM83F, which is involved in the activation of the MAPK signaling pathway 77. FAM83F is highly expressed in human PTC tissues, and normal thyroid cells overexpressing FAM83F showed loss of thyroid-specific gene expression following cross-regulation of TGF-ß and the MAPK signaling pathway with *BRAF* expression. A MEK1/2 inhibitor inhibited the expression of FAM83F with restoration of NIS expression. In the future, additional studies on the interplay between FAM83F and redifferentiation, including changes in thyroid-specific protein expression and the effect of RAI therapy, in thyroid cancers are necessary.

Conditional activation of RET/PTC1 or RET/PTC3 inhibited the expression of thyroid-specific genes promoting the differentiation process, and they also mediated the activation of the MAPK signaling pathway through Shc in thyroid cells 78. Moreover, thyroid cells expressing *RAS* or *MEK1* attenuated TSH-induced expression of *Tg* and *NIS* which was regained with MEK inhibitor treatment. These findings suggest that the MAPK signaling pathway promotes dedifferentiation in PDTC with constitutive activation of either *RAS* or RET/PTC 78. In the context of the previous study, in thyroid cells expressing RET/PTC1, MEK inhibition modulated not only NIS expression but also RAI uptake 79. Additionally, treatment with sorafenib or cabozantinib, as multikinase inhibitors (MKIs), restored RAI uptake and enhanced the expression of thyroid-specific genes in PTC cells harboring the RET/PTC1 rearrangement (Table 1) 80. Sunitinib, which is an MKI, also inhibits MEK/ERK and SAPK/JNK signaling pathways and increases the expression of thyroid-specific genes, including *NIS*, in PTC cells harboring the RET/PTC1 rearrangement (Table 1) 81. The combination of forskolin (adenylyl cyclase agonist) with sunitinib further increases the expression of thyroid-specific genes and transcription factors that bind to NUE in the cells (Table 2).

Initially, RAF and MEK inhibitors contributed to the suppression of the MAPK signaling pathway; however, this was followed by a marked reactivation of the signaling pathway to levels similar to that of controls 11. Allosteric MEK inhibitor (CH5126766 and CKI) could be an alternative candidate to overcome the reactivation of the MAPK signaling pathway. CH5126766 selectively binds to the non-phosphorylated form of MEK and stabilizes the RAF/MEK complex which is inactivated. 82. Thus, CH5126766 inhibits the feedback induction of MEK phosphorylation that occurs after ERK inhibition in cancer cells exposed to other MEK inhibitors. Nagarajah et al. reported that CH5126766, an allosteric MEK inhibitor, suppressed ERK in a sustained manner by blocking RAF reactivation, whereas selumetinib, an MEK inhibitor, suppressed ERK activation transiently in a tumor harboring the *BRAF* mutation 83. Sustained ERK inhibition via CH5126766 treatment induced higher RAI uptake and ^131^I-mediated therapeutic effects (Table 1).

Our group employed the high-throughput NIS enhancer screening platform with a dual reporter gene system to excavate two tyrosine kinase inhibitors (TKIs; K905-0266 and CTOM-DHP) which enhance NIS promoter activity 30, 84. Treatment with K905-0266 restored the expression of thyroid-specific proteins, including NIS, and promoted RAI uptake in ATC (Table 1) 84. These results were followed by an increase in RAI-mediated cytotoxicity with ^131^I by pretreating with K905-0266. Mechanism analysis revealed that K905-0266 treatment inhibited ERK phosphorylation but not AKT phosphorylation. Additionally, *in vivo* results demonstrated the effect of K905-0266 with the enhancement of the *NIS* promoter followed by increased NIS protein expression and therapeutic effects. Another excavated compound, CTOM-DHP, also influenced ATC redifferentiation with restoration of thyroid-specific protein expression followed by RAI uptake and cytotoxicity effect (Table 1) 30. Its effects were accomplished by reduction in the phosphorylation of both AKT and ERK. Moreover, nuclear imaging revealed an improvement in RAI avidity following CTOM-DHP treatment in *in vivo* study. CTOM-DHP also appeared to affect other thyroid cancers harboring *KRAS* and RET/PTC rearrangements, suggesting that they are promising candidates for restoring RAI avidity.

### PI3K/AKT signaling pathway

In addition to the MAPK signaling pathway, the PI3K/AKT signaling pathway also plays a crucial role in cell growth and proliferation and thyroid tumorigenesis (Figure 1B) 85. Several studies have reported that the stimulation of insulin-like growth factor-1 (IGF-1) inhibited iodide uptake in TSH-induced thyroid cells 86, 87. Garcia et al. reported that IGF-1 could inhibit TSH/forskolin-induced *NIS* expression through activation of the PI3K/AKT signaling pathway in thyroid cells 88. Moreover, it has been reported that inhibition of the PI3K/AKT signaling pathway could increase NIS expression and RAI uptake in thyroid cancer cells 16, 89. Specifically, PTC cells which were engineered to constitutively express NIS restored NIS expression and RAI uptake by inhibiting the PI3K/AKT signaling pathway with LY294002 as a PI3K inhibitor regardless of blockade by insulin stimulation (Table 1) 89. Interestingly, inhibition of AKT, which is downstream of PI3K, revealed that both NIS expression and RAI uptake were enhanced, exhibiting the mimicking effect of LY294002 in PTC cells. Song et al. reported that the prevalence of the *RasGRP3* mutation was higher in metastatic, RAI-refractory thyroid cancer 90. Moreover, *RasGRP3* mutation led to the promotion of cell proliferation, migration, and invasion, whereas NIS and TSHR expression and RAI uptake declined through AKT activation in thyroid cancer cells. These results suggest that an activated AKT signaling pathway may be involved in RAI refractoriness by mediating *RasGRP3* mutation in thyroid cancers (Figure 1B).

The serine-threonine protein kinase mechanistic target of rapamycin (mTOR), which is downstream of the PI3K/AKT signaling pathway, has been identified as a regulator of NIS expression, RAI uptake, and cell survival in both *in vitro* and *in vivo* studies (Figure 1B) 91. Plantinga et al. reported that the increase in the expression of thyroid-specific genes and proteins and restoration of RAI uptake through mTOR inhibition in thyroid cancer cells with the *BRAF* mutation or *PTEN* deficiency depended on TTF-1 92. mTOR acts in two functionally distinct complexes, mTOR complex 1 (mTORC1) and mTOR complex 2 (mTORC2). mTORC1 comprises mTOR, raptor, mLST8, PRAS40, and DEPTOR and regulates cell growth through effectors such as p70 S6K (also called S6K1). mTORC2 contains mTOR, rictor, mLST8, mSIN1, PROTOC, and DEPTOR and phosphorylates AKT at Ser473 and protein kinase C-ɑ (PKC-ɑ) 91. Tavares et al. reported that the role of mTOR phosphorylation as a marker of aggressiveness in PTC is closely associated with the absence of a tumor capsule, distant metastases, persistence of disease, and resistance to RAI therapy. Additionally, phosphorylation of mTOR significantly correlated with the frequency of the *NRAS* mutation but not the *BRAF* mutation 93. However, tumors with S6 phosphorylation showed less aggressive characteristics, such as the presence of a tumor capsule, absence of extrathyroidal extension, and *BRAF* wild-type, although *NRAS* mutation was significantly higher 94. Inhibition of mTORC1/mTORC2 by Torin2 treatment restored NIS expression by inhibiting the phosphorylation of AKT and S6; however, there was no effect with RAD001 treatment as an mTORC1 inhibitor in PTC (Table 1) 94. Rapamycin, an mTOR inhibitor, is effective at inhibiting the phosphorylation of S6K (mTORC1). Interestingly, treatment with rapamycin led to increased expression of thyroid-specific genes and NIS protein in thyroid cancer cells with either the *BRAF* mutation or *PTEN* deficiency but not in PTC harboring a RET/PTC rearrangement (Table 1) 92. Torin2, an mTORC1/mTORC2 inhibitor, increased *NIS* gene expression in PTC harboring the RET/PTC rearrangement instead of resistance to rapamycin treatment 94. Considering the different responses of thyroid cancers depending on cell type and mutations, further studies are needed to elucidate accurate redifferentiation mechanisms for each specific dedifferentiated thyroid cancer.

## Other signaling pathways

### Notch signaling pathway

**Figure 2:**
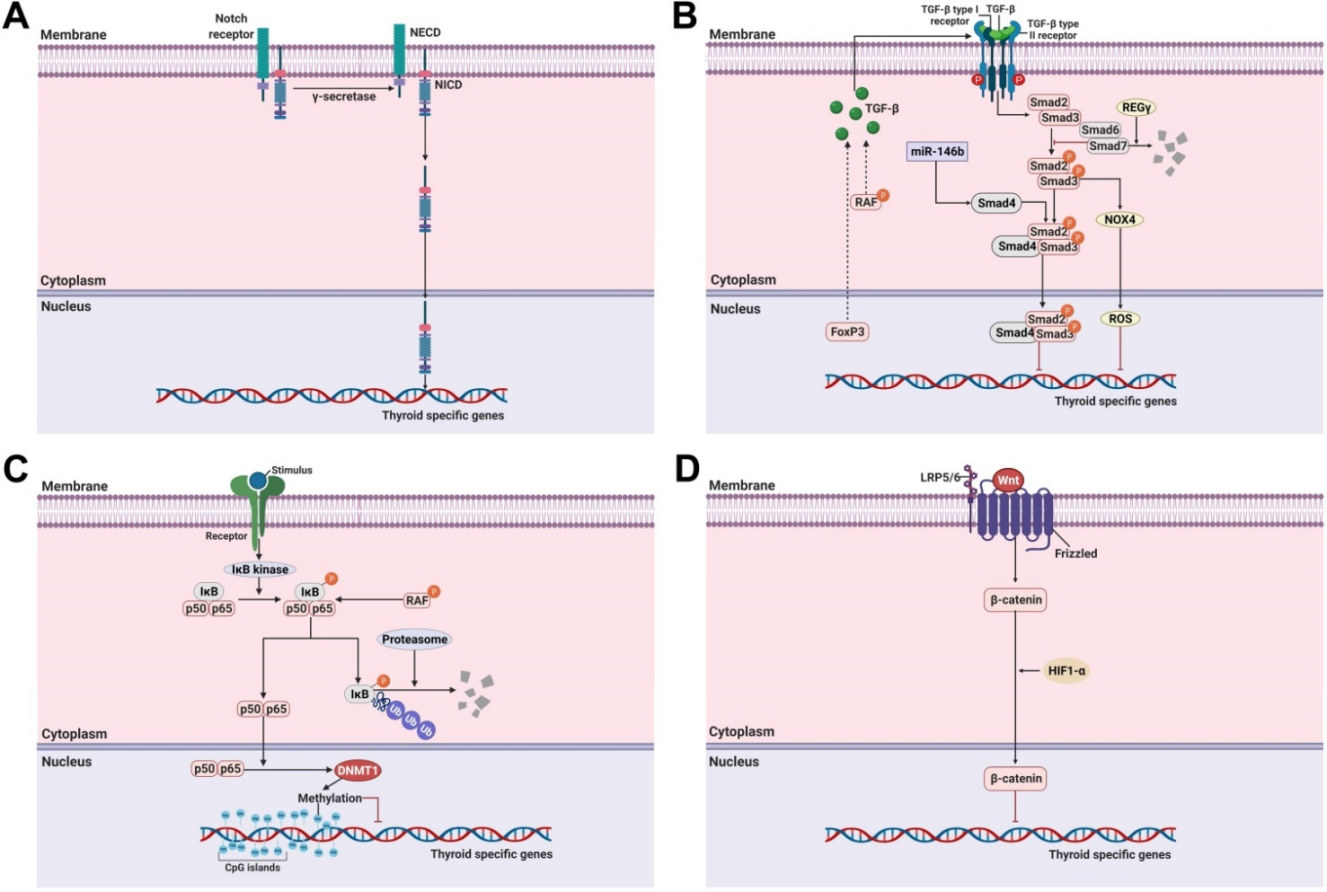
** Molecular mechanisms involved in the regulation of thyroid-specific genes including sodium iodide symporter (NIS) in RAI-refractory differentiated thyroid cancer.** (A) Notch signaling pathway. (B) TGF-ß/Smad signaling pathway. (C) Nuclear factor kappa-light-chain-enhancer of activated B cells (NF-κB) signaling pathway. (D) Wnt/ß-catenin signaling pathway. Abbreviations: DNMT1: DNA methyltransferase 1; FoxP3: forkhead transcription factor 3; HIF1-ɑ: hypoxia-inducible factor-ɑ; IκB: Inhibitory κB; LRP5/6: low-density lipoprotein receptor-related protein 5/6; NECD: notch extracellular domain; NICD: notch intracellular domain; NOX4: NADPH oxidase 4; P: phosphorylation; ROS: reactive oxygen species; Ub: ubiquitination. Images were created with BioRender.com.

Notch is a multifunctional transmembrane receptor that regulates cell differentiation, proliferation, and survival and exerts oncogenic or tumor suppressor effects depending on cell types 95. The Notch signaling pathway comprises 4 Notch receptors (Notch1-4) and 5 ligands (δ-like 1, 3, and 4, Jagged-1, and Jagged-2). Once Notch receptors are activated by binding to ligands, they promote two sequential proteolytic cleavages via the ɣ-secretase protease complex 96. This results in the release of a cytoplasmic domain fragment (the second cleavage occurs within the transmembrane area) which moves from its transmembrane location into the nucleus and modulates gene expression (Figure 2A).

Ferretti et al. reported the correlation between the Notch signaling pathway and the differentiation of thyroid cancers from normal thyroid cells 95. Overexpression of the Notch signaling pathway, including that of Notch1, Notch3, and Hes1, is directly associated with a differentiation phenotype (*NIS*, *TPO*) expression of both thyroid cancers and TSH-stimulated normal thyroid cells. Subsequently, Carre et al. reported the importance of Hes1 during mouse thyroid development and differentiation as with NIS 97. Other studies also reported the importance of the Notch signaling pathway in the differentiation of thyroid cancers 96, 98. Yu et al. identified that resveratrol, as a Notch1 activator, could restore NIS expression and thyroid-specific transcription factors (Table 1) 96. Somnay et al. found that repressed Notch3 expression was inversely correlated with tumor size, extrathyroidal extension, and distant metastasis following dedifferentiation in clinicopathological features 98. Overexpression of Notch intracellular domain 3 (NICD3), which is cleaved from the Notch receptor and then translocated from the membrane to the nucleus to regulate target genes, in FTC cells led to the functional activation of the genes involved in downstream signaling pathways such as *Hes1*, *HEY1*, and *HEY2*. Additionally, the gain of function of FTC cells with NICD3 overexpression induced the expression of thyroid-specific genes. In contrast, suppression of Notch3 by siRNA could silence the expression of thyroid-specific genes in thyroid cancers. These results suggest that the activation of Notch1 and Notch3 is a potential strategy for the redifferentiation of thyroid cancers.

### TGF-ß/Smad signaling pathway

The TGF-ß signaling pathway regulates cellular functions such as proliferation, differentiation, adhesion, apoptosis, and survival in multiple cancers, including thyroid cancer 23, 99. TGF-ß is a secreted protein with three isoforms, i.e., TGF-ß1, TGF-ß2, and TGF-ß3 100. Their effects are initiated by binding to a type II receptor on the cell membrane. The TGF-ß-type II receptor complex then recruits a type I receptor 101. This new complex stimulates the phosphorylation of Smad2 and Smad3 (R-Smad) which is then combined with Smad4 (Co-Smad) This complex subsequently enters the nucleus where it participates in the regulation of gene expression. Inhibitory Smads comprise Smad6 and Smad7 which oppose the activation of the TGF-ß signaling pathway (Figure 2B) 101.

Several reports have noted that TGF-ß plays a critical role in the growth and differentiation of normal thyroid cells 100-103. Costamagna et al. reported that TGF-ß treatment decreases the expression and interaction of PAX-8 with Smad3 in response to TGF-ß stimulation, and PAX-8 decreases the DNA-binding activity of PAX-8 in normal thyroid cells 23. Although inhibition of the MAPK signaling pathway, constitutively activated by *BRAF*, was considered a breakthrough, it is not always effective in resistant tumors, including RAI-refractory PTC. This suggests that other compensatory mechanisms affect adaptive resistance development in *BRAF*. Riesco-Eizaguirre et al. demonstrated that the *BRAF* oncogene activates the secretion of an autocrine TGF-ß loop leading to MEK-ERK-independent NIS diminution which operates in tandem with the MEK-ERK pathway in thyroid cancer (Figure 2B) 22. Additionally, they noted that high TGF-ß expression is closely associated with extrathyroidal extension, nodal metastasis, and *BRAF* status in human PTC samples. Azouzi et al. reported that NADPH oxidase 4 (NOX4) expression is regulated by a *BRAF* mutation via the TGF-ß/Smad signaling pathway and that NOX4-dependent reactive oxygen species (ROS) production plays a critical role in the reduction of NIS expression in *BRAF*-mutated PTC (Figure 2B) 104.

Forkhead transcription factor 3 (FoxP3) is a key regulator of CD4^+^CD25^+^ regulatory T cells in maintaining immunologic tolerance 105, 106. Its positive expression is associated with immune evasion of cancers, possibly by inducing the secretion of immunosuppressive cytokines such as TGF-ß1 and IL-10. Importantly, FoxP3 expression was correlated with extrathyroidal invasion, distant metastasis, and resistance to RAI therapy in PTC 107, 108. Based on these findings, Ma et al. proposed that FoxP3 inhibits NIS expression by inducing TGF-ß1 secretion and subsequent activation of phosphorylation of Smad3 in thyroid cancer (Figure 2B) 108.

Recently, REGɣ, a proteasome activator, has been shown to enhance dedifferentiation by degrading Smad7, resulting in the activation of the TGF-ß/Smad signaling pathway in ATC with resistance to RAI therapy (Figure 2B) 109.

### Platelet-derived growth factor receptor-ɑ

As noted previously, mTOR inhibition promotes the expression of thyroid-specific genes and proteins and RAI uptake with TTF1-dependent redifferentiation in thyroid cancers 92. Lopez-Campistrous et al. reported the inverse relationship between platelet-derived growth factor receptor-ɑ (PDGFRɑ) and TTF-1 expression in PTC 110. PDGFRɑ activation disrupts the transcriptional activity of TTF-1 and dephosphorylates TTF-1, resulting in its translocation from the nucleus to the cytoplasm. This phenomenon leads to a reduction in the expression of thyroid-specific genes and uptake of RAI in PTC. This finding suggests that PDGFRɑ blockade improves RAI therapy.

### NF-κB pathway

The nuclear factor kappa-light-chain-enhancer of activated B cells (NF-κB) signaling pathway plays a pivotal role in the regulation of inflammatory responses involved in oncogenesis 111. Most NF-κB dimers are sequestered in the cytoplasm in an inactive state by interaction with inhibitory κBs (IκBs) when not stimulated (Figure 2C). In response to stimulation, such as with lipopolysaccharide (LPS), tumor necrosis factor-ɑ (TNF-ɑ), and LTβR, degradation of IκBs is initiated by phosphorylation, ubiquitination, and degradation via the IκB kinase (IKK). NF-κB is then activated, resulting in its translocation to the nucleus 112.

Reale et al. highlighted the role of NF-κB in thyroid physiology by noting that genetic deletion of the NF-κB essential modulator locus could promote the downregulation of thyroid-specific genes (*NIS*, *TPO*, *Tg*, *PAX-8*, and *TTF-1*), resulting in the development of hypothyroidism 113. Additionally, several articles reported that the NF-κB signaling pathway is related to the function and homeostasis of thyroid cells 24, 25, 114, 115. In detail, NF-κB, especially the p65 subunit, mediates the effect of LPS to increase expression of TSH-induced Tg, NIS, and TPO and RAI uptake in normal thyroid cells. However, unlike LPS stimulation, TNF-ɑ stimulation downregulated NIS expression through an increase of IκB-ɑ in normal thyroid cells 28, 116. One possible implication of the antagonistic effects between LPS and TNF-ɑ stimulation for NIS expression is that the intervention of additional signaling pathways may mediate the affinity of NF-κB transcription factors for other promoters. Elucidation of the exact molecular mechanisms of NIS expression by the NF-κB signaling pathway is still required for better understanding of thyroid physiology.

Increased NF-κB activation in thyroid cancer cell lines and human thyroid cancer tissues has been documented 117-119. Moreover, this phenomenon leads to increased cell proliferation, growth, and resistance to drug-induced apoptosis through anti-apoptotic signaling pathways.

Starenki et al. initially reported that NF-κB inhibition could potentiate the therapeutic effect of ionizing radiation in thyroid cancer in *in vitro* and *in vivo* studies 120. However, several reports on the effect of NF-κB inhibition to reinduce RAI uptake and therapy found no promising results 121, 122. Meng et al. reported that ^131^I rather induced NF-κB activation followed by interruption of the therapeutic efficiency of RAI therapy 121. Additionally, another study described that the cell survival rate declined following treatment with a combination of NF-κB gene silencing and ^131^I 122. However, there was no improvement in RAI uptake via NF-κB inhibition. Finally, inhibition of NF-κB is not sufficient to enhance RAI uptake, and there are no results documenting a change in thyroid-specific gene expression. Therefore, in the future, it is necessary to consider other factors or signaling pathways that interact with the NF-κB signaling pathways with verification of changes in thyroid-specific gene expression in thyroid cancers.

Choi et al. reported that upregulated DNA methyltransferase 1 (DNMT1) via NF-κB activation caused by *BRAF* mutation suppresses the NIS expression caused by its promoter methylation (Figure 2C) 123. In this manner, it is possible to infer that the interplay of other factors is involved in the activation of NF-κB, and overcoming this situation may be a better treatment strategy when weaker RAI uptake via NF-κB inhibition occurs.

### Wnt/ß-catenin signaling pathway

The role of the Wnt/ß-catenin signaling pathway in the regulation of cell growth and proliferation and constitutive activation resulting in the development of cancer has been well-established 29. After the canonical Wnt ligand binds to the Frizzled receptor and LRP5/6 as a co-receptor, ß-catenin is activated and translocated from the cytoplasm to the nucleus where it promotes the expression of various genes (Figure 2D).

Sastre-Perona et al. reported that Wnt-independent ß-catenin regulates cell proliferation and differentiation in normal thyroid cells 124. Additionally, they elucidated that the direct interaction of ß-catenin with PAX-8 increases its transcriptional activity for *NIS* expression. These results suggested that ß-catenin is a positive regulator of NIS and RAI uptake under physiological conditions.

In contrast, it has been demonstrated that aberrant ß-catenin expression in the nucleus correlates with the dedifferentiation of thyroid cancers 125, 126. Lan et al. demonstrated that HIF1-ɑ-induced ß-catenin modifies NIS subcellular localization and impairs RAI uptake in FTC cells (Figure 2D) 127. Suppression of ß-catenin reversed these changes with RAI therapy and regulation of NIS localization in HIF1ɑ-overexpressing FTC cells.

## Epigenetic alterations

### DNA methylation

**Figure 3:**
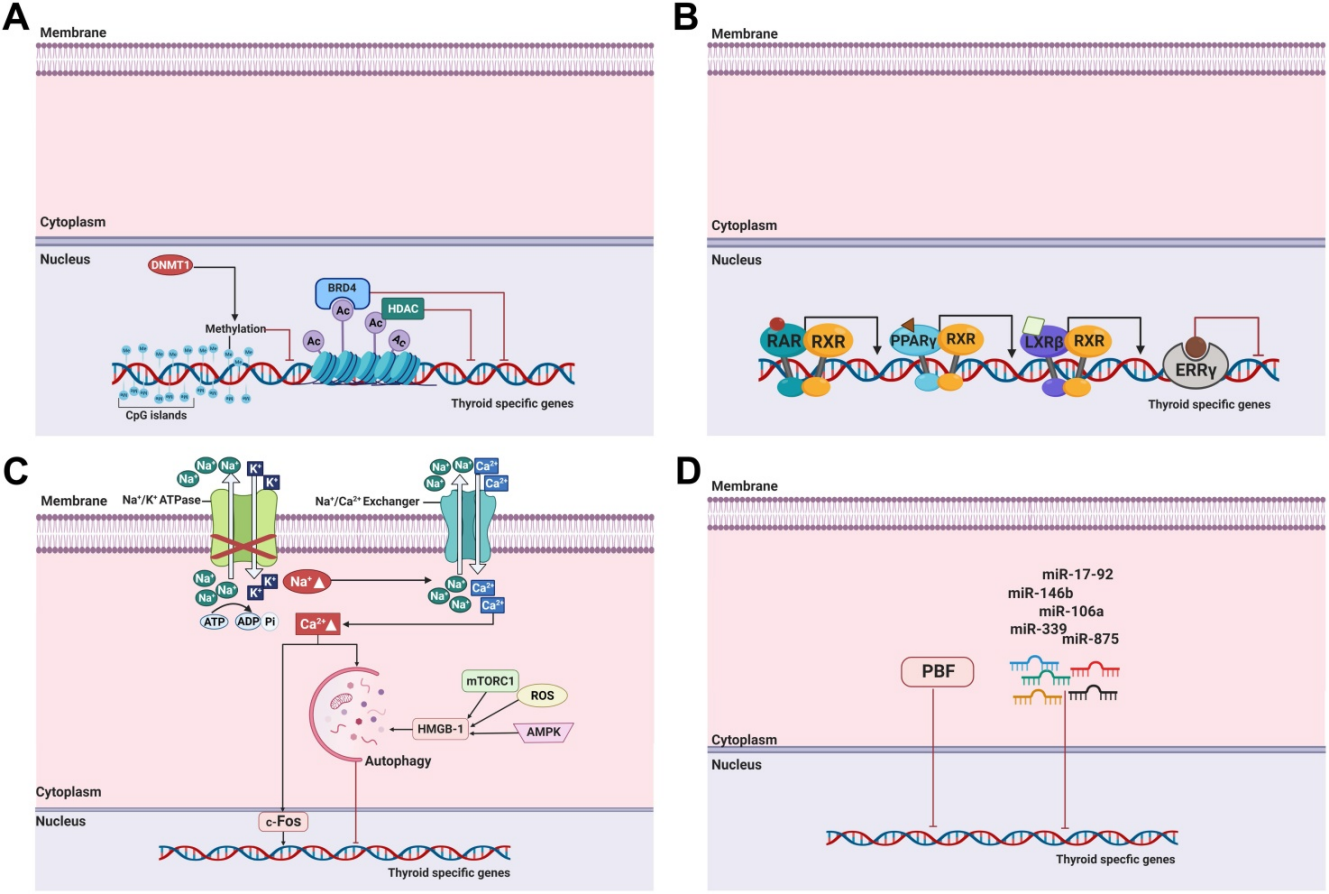
** Molecular mechanisms involved in the regulation of thyroid-specific genes, including NIS in RAI-refractory differentiated thyroid cancer.** (A) Epigenetic alterations, including DNA methylation and histone modification, and bromodomain-containing protein 4 (BRD4). (B) Nuclear receptors RAR, PPARɣ, LXRß and ERRɣ. (C) Autophagy. (D) MicroRNAs and PBF. Abbreviations: Ac: acetylation; BRD4: bromodomain-containing protein; DNMT1: DNA methyltransferase 1; ERRɣ: estrogen-related receptor ɣ; HDAC: histone deacetylase; HMGB1: high mobility group box 1**;** LXRß: liver X receptor ß; miR: microRNA; mTORC1: mTOR complex 1; PBF: pituitary tumor-transforming gene 1 (PTTG1)-binding factor; PPARɣ: peroxisome proliferator-activated receptor ɣ; RAR: retinoic acid receptor; ROS: reactive oxygen species; RXR: retinoid X receptor. Images were created with BioRender.com.

DNA methylation, one of the most commonly occurring epigenetic events, is the hallmark of human cancers, including thyroid cancer 29. It involves covalent chemical modification, resulting in the addition of a methyl group (CH_3_) to the carbon residue at the 5'-position of the cytosine ring. DNA methylation typically occurs in CpG dinucleotides (Figure 3A) 128.

Previous studies demonstrated that aberrant NIS hypermethylation occurs in thyroid cancer regardless of cell type 129-131. One study reported that the methylation level and frequency between benign and malignant thyroid tumors did not differ, and high NIS promoter methylation levels led to reduced *NIS* expression 131. Later, Galrão et al. identified new CpG islands in the NIS proximal promoter by *in silico* screening and demonstrated that *NIS* hypermethylation had a significant inverse correlation with *NIS* messenger RNA (mRNA) expression 132. Aberrant hypermethylation of thyroid-specific genes, namely, *TSHR*, *Pendrin*, and thyroid-specific transcription factors such as *TTF-1*, were also discovered in thyroid cancers 133, 134. The application of DNA demethylating agents, such as 5-azacytidine and its deoxy derivative (5-aza-2'-deoxycytidine), to the aberrant NIS hypermethylated thyroid cancer restored RAI uptake following NIS expression (Table 1) 132, 135.

The *BRAF* mutation and its relation to aberrant gene methylation in thyroid cancer could be one of the possible targets for restoring the expression of thyroid-specific genes 136. Khan et al. noted that the frequency of hypermethylation of the *TSHR* gene promoter was 73.3% in patients with *BRAF* mutation, whereas it was only 8.8% in patients with wild-type *BRAF* 137. Liu et al. demonstrated the relationship between *TSHR* promoter methylation and the MAPK signaling pathway in thyroid cancer. In this study, the extent of *TSHR* promoter methylation was decreased by MEK inhibitor treatment, suggesting that the process for restoring the expression of thyroid-specific genes was linked with gene methylation 56. Choi et al. reported that the *BRAF* mutation in thyroid cancer inhibits *NIS* expression by upregulating DNMT1, an inducer of promoter methylation in CpG islands (Figure 1A) 123.

### Histone modification

Histone modifications, such as acetylation, methylation, phosphorylation, and ubiquitination, are critical determinants of the epigenetic state in addition to DNA methylation 138. A nucleosome comprises 147bp of DNA wrapped around an octamer of histones H2A, H2B, H3, and H4 85. Histone modification can lead to either gene activation or inhibition depending on which residues are modified and the type of modification 139. Overall, histone modification plays an important role in gene regulation and other chromatin-based processes 138.

Among histone modifications, histone acetylation typically occurs at lysine residues in the N-terminal tails of H3 and H4 by histone acetyltransferases that catalyze these processes. This leads to an open chromatin structure, resulting in the promotion of gene expression. Conversely, histone deacetylation is catalyzed by HDACs, resulting in the condensation of chromatin and then repression of gene expression 140. Importantly, dysregulated histone acetyltransferase and HDAC activation are closely related to growth, proliferation, and differentiation of cells in cancers (Figure 3A) 138.

HDAC inhibitors may not only act specifically against limited types of HDACs (HDAC isoform-selective inhibitors) but also against all types of HDACs (pan-inhibitors). HDAC inhibitors are classified based on their specificity as members of five classes of compounds: (1) hydroxamic acids (hydroxamates), (2) short chain fatty (aliphatic) acids, (3) benzamides, (4) cyclic tetrapeptides, and (5) sirtuin inhibitors such as the pan-inhibitor nicotinamide and the specific SIRT1 and SIRT2 inhibitors sirtinol and cambinol, respectively 141.

Puppin et al. reported that modification of histone acetylation occurs in thyroid tumors 142. They noted that the level of acetylated lysines 9 and 14 in H3 (H3K9-K14ac) was higher in follicular adenomas, PTC, FTC, and undifferentiated carcinomas than in normal tissue, and no difference in the H3 histone (H3K18ac) was found between normal tissue and undifferentiated carcinoma. It was suggested that modifications in histone acetylation is an early event in thyroid tumorigenesis, and acetylation of H3K18ac is switched off in the transition from differentiated to undifferentiated thyroid tumors.

Kitazono et al. were the first to demonstrate that treatment with depsipeptide (romidepsin, FR901228), a class I HDAC inhibitor, restored NIS expression and RAI uptake 143. This suggests its utility as an HDAC inhibitor at *in vitro* levels (Table 1). Based on the results of this study, other preclinical studies have indicated that RAI avidity improved after reinduction of thyroid-specific gene expression and NIS localization to the cell membrane following treatment with the HDAC I and II inhibitors valproic acid, panobinostat (LBH589), and entinostat in *in vitro* and *in vivo* studies (Table 1) 144-150. The use of pan-inhibitors such as vorinostat (suberoylanilide hydroxamic acid [SAHA]), trichostatin A, and sodium butyrate in thyroid cancer also resulted in the redifferentiation of cancer after enhanced thyroid-specific gene expression, leading to RAI accumulation (Table 1) 16, 146, 150, 151. Jang et al. developed a new analog, HDAC inhibitor (AB1 to AB13), and excavated the effective compounds (AB2, AB3, and AB10) 152. This allowed NIS, *PAX-8*, *TTF-1*, *TTF-2*, and *TSHR* gene expression to promote redifferentiation of thyroid cancers (Table 1).

Recently, oncogene-activated signaling pathways have been reported to also control histone posttranslational modification that affects the expression of thyroid-specific genes 153. This finding supports attempts to convert dedifferentiated thyroid cancer into redifferentiated thyroid cancer by modulating histone acetylation and deacetylation.

Among the studies mentioned previously, Hou et al. reported the synergistic effects of vorinostat with MAPK, mTOR, and AKT inhibitors to upregulate thyroid-specific gene expression and RAI accumulation in thyroid cancers (Table 2) 16. In addition, the same group demonstrated that *BRAF*-activated NIS silencing could be influenced by histone deacetylation at critical regulatory regions of the *NIS* promoter 140. Cheng et al. also demonstrated that combination treatment of thyroid cancer cells with vorinostat and vemurafenib, a BRAF inhibitor, synergistically induced NIS expression and localization to the cell membrane and RAI uptake (Table 2) 19. Remarkably, TSH stimulation during this treatment regimen revealed dramatic effects compared with treatment without TSH 16, 19. Additionally, Fu et al. demonstrated that panobinostat, an HDAC inhibitor, induced a more robust redifferentiation when combined with selumetinib (a MEK inhibitor) or dabrafenib (a BRAF inhibitor) treatment regardless of *BRAF* mutation status (Table 2) 154. Notably, tributyrin, a butyrate analog, is a trimer of butyric acid with retinoic acid (RA) that enhances RAI uptake by upregulating NIS expression in PDTC (Table 2) 155. Moreover, combination treatment with DNA demethylating agents and HDAC inhibitors were used to increase thyroid-specific gene expression and restore RAI therapy effectiveness in thyroid cancer (Table 2) 156, 157. Provenzano et al. reported that concurrent treatment of thyroid cancer cells with an HDAC inhibitor (sodium butyrate) and a DNA demethylating agent (5-aza-2'-deoxycytidine) promoted an increase in NIS expression and RAI uptake 156. In the case of treatment with a single agent, although it may increase *NIS* expression, it did not affect RAI uptake owing to altered posttranslational modification. Another study also identified the effect of combination treatment with an HDAC inhibitor (valproic acid) and a DNA demethylating agent (5-aza-2'-deoxycytidine) to increase RAI uptake 157.

Recently, Fu et al. reported that dual inhibition of the MAPK signaling pathway and enhancement of zeste homolog 2 (EZH2) as a histone methyltransferase (HMT) had a synergistic effect in the redifferentiation of PTC harboring the *BRAF* mutation (Table 2) 158. Specifically, the use of an MEK inhibitor (selumetinib) or a BRAF inhibitor (dabrafenib) with an EZH2 inhibitor (tazemetostat) improved thyroid-specific protein expression and enhanced the effect of RAI therapy.

## Nuclear receptors

### Retinoic acids

Retinoic acids (RAs), retinol, and their derivatives, are related to metabolites of vitamin A and act on the nuclear receptors RA receptor (RAR) and retinoid X receptor (RXR) (Figure 3B) 159. There are three groups of RAs. The first group includes retinol, retinal, RA, and isotretinoin; the second group includes etretinate and acitretin; and the third group includes tazarotene and bexarotene 159.

Several studies have noted a correlation between treatment with RAs and differentiation of thyroid cancer cells 160-162. Type I iodothyronine 5'-deiodinase activity, a differentiation marker, increased after treatment with RAs for thyroid cancer, whereas that of CD97, a dedifferentiation marker, decreased.

NIS expression in normal thyroid cells has been shown to decrease after RA treatment 163. However, most thyroid cancers, such as FTC, PTC, and ATC, exhibit an elevation in NIS expression and NIS-mediated RAI uptake after RA treatment (Table 1) 148, 163-166. Among these reports, Jeong et al. demonstrated that treatment of thyroid cancer with RA alters the expression of several genes involved in cell growth and differentiation and increased NIS and RAI uptake in a microarray experiment 166.

### Peroxisome proliferator-activated receptor ɣ

Peroxisome proliferator-activated receptor (PPAR) is a nuclear receptor superfamily and comprises three forms: PPARɑ, PPARß, and PPARɣ 4. It is involved in the regulation of cell proliferation and redifferentiation and is a ligand-dependent transcription factor that must form a heterodimer with RXR to bind to their response elements and activate gene expression (Figure 3B). The rearrangement of PAX-8/PPARɣ primarily occurs in FTC and follicular-variant PTC 167.

Studies have shown that the application of a PPARɣ agonist can cause redifferentiation in thyroid cancers 148, 168, 169. Fröhlich et al. demonstrated the action of several thiazolidinediones, such as rosiglitazone, troglitazone, and pioglitazone, for redifferentiation, proliferation, and apoptosis in FTC (Table 1) 168. In addition, Park et al. reported that treatment with the PPARɣ agonist troglitazone could upregulate NIS expression, whereas CD97, a marker of thyroid dedifferentiation, downregulated its expression. This suggests that PPARɣ agonists induce redifferentiation in different types of thyroid cancer (Table 1) 169.

### Liver X receptors

Liver X receptors (LXRs) are members of the nuclear receptor family and are ligand-activated transcription factors (Figure 3B) 170. LXRs exist as two isoforms: LXRɑ and LXRß, and they have a critical role in cholesterol transport, glucose and lipid metabolism, regulation of immune response, and cell differentiation and growth 171. Additionally, the effects of LXRs have been studied as potential targets for cancer therapeutics 170.

Dendrogenin A (DDA), a class of LXR ligand, is a mammalian cholesterol-derived metabolite displaying the characteristics of a tumor suppressor 172. Bauriaud-Mallet et al. reported the possibility of DDA inducing redifferentiation in thyroid cancer (Table 1) 173. DDA and GW3965, a canonical LXR ligand, treatment could induce redifferentiation and enhanced thyroid-specific protein expression and RAI uptake in thyroid cancers.

### Estrogen-related receptors

Estrogen-related receptors (ERRs) are an active nuclear receptor family and consist of three isoforms: ERRɑ, ERRß, and ERRɣ. They play an important role in disease development in cancer and metabolic disorders (Figure 3B) 174.

Singh et al. first demonstrated the effect of the ERRɣ inverse agonist GSK5182 to improve RAI therapy followed by an increase in membrane-localized NIS expression and RAI uptake (Table 1) 175. These effects were accompanied by the downregulation of ERRɣ and activation of the ERK/MAPK signaling pathway. Recently, new ERRɣ inverse agonists have also shown efficacy at increasing RAI avidity in both *in vitro* and *in vivo* studies (Table 1) 176, 177.

## Other factors

### Autophagy

Autophagy is one of the important biological processes for cancer development and progression 178. It may be triggered by hypoxia, nutritional deprivation, DNA damage, and other factors to facilitate the degradation of cytoplasmic materials such as damaged organelles and misfolded proteins 179. These degraded materials are then engulfed into autophagosomes with a double membrane structure and degraded by fusion with lysosomes into autolysosomes, with the purpose of homeostasis maintenance and survival for cell recycling and various other stressors within the cell 178, 179. Lin et al. suggested autophagy as a new target for thyroid cancer therapy 180. Treatment with doxorubicin and irradiation exposure for thyroid cancer inhibited cell survival; however, combination treatment with 3-methyladenin, an autophagy-specific inhibitor, triggered chemoresistance and irradiation resistance. Those resistances were reversed by treatment with the autophagy activator RAD001 that sensitizes thyroid cells to doxorubicin and irradiation exposure. Moreover, they determined that the effects of autophagic activation are modulated mainly by Met inhibition 181. Plantinga et al. reported that autophagic activity is associated with membranous NIS expression and response to RAI therapy in patients with non-MTC 179. The same group then noted that upregulation of NIS expression and restoration of RAI uptake were mediated by autophagy-activating compounds, known as digitalis-like compounds (DLCs), by the accumulation of intracellular Ca^2+^ and FOS activation (Figure 3C, Table 1) 182. Interestingly, DLCs induced redifferentiation with enhanced NIS expression in several thyroid cancer cell lines irrespective of mutations such as *BRAF*, *PTEN* loss, or RET/PTC rearrangement. Based on the preceding research showing that DLCs had a potent effect on redifferentiation in RAI-refractory PTC, the effects of DLCs in ATC were also demonstrated with increased NIS expression and RAI uptake activity although autophagy activity was lower than in PTC 183. Recently, Chai et al. noted the function of the high mobility group box 1 (HMGB1), a nuclear protein, that plays a role in autophagy-mediated NIS degradation via ROS, AMPK, and mTOR signaling pathways (Figure 3C) 184. Preclinical studies examined the association of HMGB1 with clinical pathological features. The results indicated that tumor size, lymph node metastasis, and clinical stage are closely connected with high HMGB1 expression. The results of these studies suggest that the activation of autophagy serves as a promising therapeutic option to restore RAI avidity and to increase therapeutic response to ^131^I.

### Bromodomain-containing protein 4

Bromodomain-containing protein 4 (BRD4) is an epigenetic regulator and a member of the bromodomain and extra-terminal domain families. It plays a critical role in the genesis and development of various diseases, including cancer 185. It affects gene transcription by binding to acetylated histones 186.

Gao et al. evaluated the degree of BRD4 expression levels in thyroid cancers and the possibility of BRD4 inhibition for cell viability, NIS expression, and RAI uptake (Figure 3A) 186. Primarily, BRD4 was overexpressed in PTC specimens compared with that in normal tissues from patients and cell lines, suggesting the function of BRD4 in cancer progression. Treatment of thyroid cancer cells with JQ1, which inhibits the acetyl-lysine recognition site of BRD4, enhances NIS expression and RAI uptake and apoptosis (Table 1).

### MicroRNAs

MicroRNAs (miRNAs) are small, non-coding RNAs containing approximately 19-25 nucleotides. They regulate gene expression by binding to the 3'-untranslated regions of mRNA at the posttranscriptional level and blocking the translation or cleaving targeted mRNA 187, 188. A single miRNA can bind to numerous gene mRNAs; therefore, the regulation of miRNA in mRNA plays a critical role in many biological functions 189. In thyroid cancer, miRNA is important for cancer initiation and progression, and it may serve as a diagnostic and prognostic biomarker.

To date, several miRNAs have been excavated to modulate NIS expression and RAI accumulation in both normal thyroids and cancers (Figure 3D). Among these miRNAs, miR-146b is one of the most studied miRNAs in thyroid cancer. Riesco-Eizaguirre et al. suggested that miR-146b, which is overexpressed in patients with PTC, is a key strategy for the conversion from dedifferentiation to differentiation by regulating the miR-146b/PAX-8/NIS circuit for the reinduction of RAI accumulation 190. miR-146b expression was negatively regulated by HDAC3, and miR-146b antagonists have been shown to reinduce the expression of thyroid-specific genes (*NIS*, *TSHR*, and *TPO*) followed by an increase in RAI uptake in dedifferentiated FTC 191. Apart from NIS, other thyroid-specific genes such as iodothyronine deiodinase 2 (*DIO2*), which converts T4 into T3, and iodotyrosine dehalogenase 1 (*DEHAL1*), which controls the recycling of iodide for thyroid hormone synthesis, were also inversely regulated by miR-146b 190, 192. Additionally, Geraldo et al. reported that disruption of the TGF-ß/Smad4 signaling pathway by overexpression of miR-146b could deregulate the expression of the thyroid-specific genes *NIS*, *Tg*, *TPO*, and *TSHR* 193. Recently, Hou et al. demonstrated the miR-146b could modulate NIS expression with translocation to the membrane by targeting MUC20 through the MET signaling pathway in dedifferentiated thyroid cancer 194. Therefore, the importance of miR-146b regulation as a potential strategy for cell redifferentiation, restoration of RAI uptake, and modulation of several signaling pathways is clear.

Ricarte-Filho et al. reported the role of miR-let-7 as a positive regulator for the thyroid-specific genes TTF-1 and Tg by inhibiting MAPK activation in PTC harboring the RET/PTC rearrangement; however, NIS expression was not detected even with miR-let-7f overexpression 188. In patients' samples, hsa-let-7f was found to be generally overexpressed in thyroid cancers regardless of type, whereas has-let-7b expression patterns differed among thyroid cancer types 195. This suggests it is a valid diagnostic biomarker for the classification of PTC and ATC.

Shen et al. assessed serum miRNA profiles in patients with PTC having non-RAI-avid or RAI-avid lung metastasis and found that miRNA-106a was upregulated in patients with PTC with non-RAI-avid lung metastasis 196. Furthermore, they found that miRNA-106a directly targeted RARß and could regulate NIS and TSHR expression and RAI uptake in thyroid cancers, suggesting miRNA-106a as a new diagnostic and therapeutic target.

In addition to the previously mentioned miRNAs, miR-339, miR-875, and miR-17-92 have been found to improve RAI uptake and NIS expression by modulating the upregulated miRNA in thyroid cancers 197, 198.

Recently, the effect of TKI on the expression of miRNAs has been assessed in thyroid cancer cells 199. Treatment with selumetinib, a MEK inhibitor, increased NIS expression and downregulated both hsa-let-7f and miR-146b expression in PTCs, whereas ATCs only produced a partial response.

### Others

As a non-coding RNA group, the role of SLC6A9 long non-coding RNA was also used to convert RAI resistance into RAI sensitivity in PTC via PARP-1 activity, suggesting that SLC6A9 is essential to maintain RAI sensitivity 200. However, the change of thyroid-specific genes and protein expression was not assessed in this study.

Dong et al. suggested the role of non-nucleoside reverse transcriptase inhibitors for the redifferentiation of thyroid cancer 201. In ATC, nevirapine, a non-nucleoside reverse transcriptase inhibitor, upregulated the expression of NIS and TSHR, and TSH co-stimulation further increased *NIS* mRNA expression. However, the improvement in RAI uptake was disappointing (Table 1). Recently, the same group reported the effect of nevirapine on dedifferentiated thyroid cancer for conversion to a redifferentiated status (Table 1) 17. The thyroid-specific genes *TSHR*, *PAX-8*, and *NIS* were significantly upregulated, and improved RAI uptake was noted, whereas expression of CD97, an undifferentiation marker, declined in dedifferentiated thyroid cancers. Moreover, cytoplasmic and membrane fraction expression of NIS increased with the latter being more effective. Interestingly, it was increased by the TSHR/cAMP/cAMP response element-binding protein (CREB)/PAX-8 signaling pathway, suggesting the therapeutic potential of non-nucleoside reverse transcriptase inhibitors to restore RAI uptake.

AXL is a tyrosine kinase receptor that regulates cell proliferation, survival, invasiveness, and chemoresistance in several cancers. AXL stimulation via its ligand triggers multiple signaling pathways, such as MAPK, PI3K/AKT, Jak/Stat, and NF-κB 202. Collina et al. demonstrated that elevated expression of AXL closely correlated with RAI refractoriness and disease persistence or recurrence in PTCs and a decrease in NIS expression and iodide uptake in an *in vitro* model 203. Additionally, the concurrent presence of the *BRAF* mutation and high AXL expression significantly correlated with the frequency of RAI refractoriness and disease recurrence in patients with PTC.

## Membrane targeting of NIS

**Figure 4:**
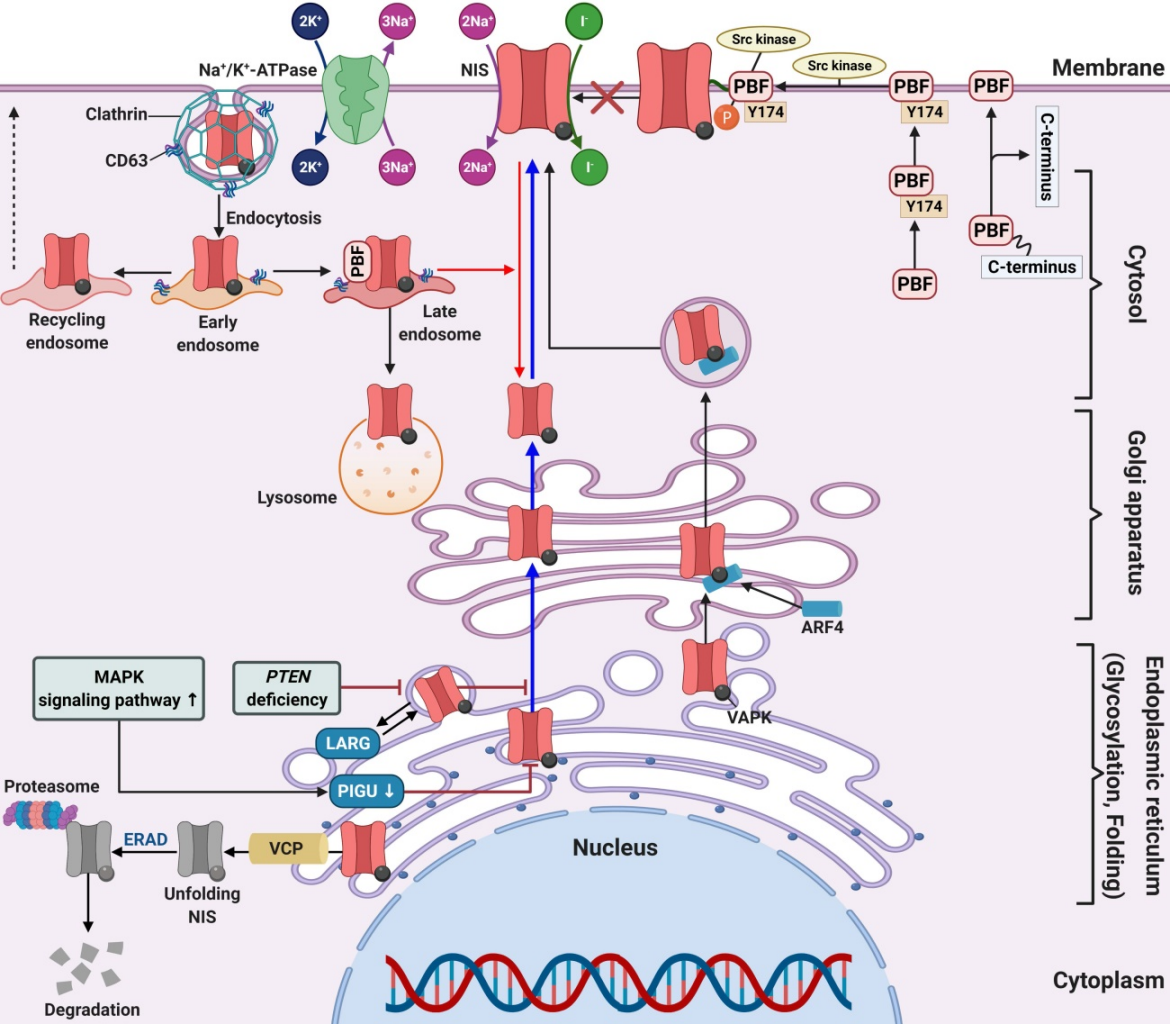
** Schematic diagram of factors related to NIS membrane targeting in RAI-refractory differentiated thyroid cancer.** Abbreviations: ARF4: ADP-ribosylation factor 4; ERAD: endoplasmic-reticulum-associated protein degradation; LARG: leukemia-associated RhoA guanine exchange factor; PBF: pituitary tumor-transforming gene 1 (PTTG1)-binding factor; PIGU: phosphatidylinositol glycan anchor biosynthesis class U; VCP: valosin-containing protein. Image was created with BioRender.com.

Membrane protein is produced in the endoplasmic reticulum and then moves to the cellular membrane via the golgi complex 204. Posttranslational modification of proteins, such as phosphorylation and glycosylation, is required for the process of membrane localization of proteins (Figure 4) 205.

In previous studies, immunohistochemistry of patient samples revealed that approximately 70%-80% of thyroid cancers expressed or even overexpressed NIS 206, 207. However, expression was primarily in the cytoplasm and not targeted to the plasma membrane. Regarding posttranslational regulation, impairment of NIS function is not limited to decreased or absent expression, but may be the result of impaired membrane targeting of NIS and insufficient retention of NIS in the membrane 4, 159. Therefore, NIS must be expressed, targeted, and maintained in the plasma membrane of thyroid cells for proper iodide transport.

The tumor microenvironment (TME) includes vessels, immune cells, fibroblasts, signaling molecules, and extracellular matrix 208. Tumors can affect the TME by releasing extracellular signals which promotes angiogenesis of the tumor and induces peripheral immune tolerance 209. These signals can induce a change in the TME, and conversely, the TME can influence the growth or metastasis of tumors. Eventually, a tumor's heterogeneity will naturally increase owing to the various factors existing in the TME. Recently, a study highlighted that changes in the TME such as hypoxia or quiescence could impair NIS expression at the plasma membrane and induce distinct patterns of NIS subcellular localization followed by a reduction in NIS-mediated RAI uptake in exogenous *NIS*-expressing cancer cells 210. These results suggest that there are diverse underlying molecular mechanisms that bring about change to NIS subcellular localization. Although this study used non-thyroid cancer cells to investigate the molecular mechanisms underlying impairment of NIS membrane targeting in a hypoxic and quiescent environment, the authors postulated that NIS localization on the plasma membrane could be impaired in DTCs by similar molecular mechanisms. Lan et al. reported that the overexpression of HIF1-ɑ, an hypoxia factor, could influence expression and localization of NIS followed by a decline of RAI uptake in FTC 127. Therefore, appropriate understanding of the TME is also needed to affect the RAI refractoriness.

In a series of 60 human PTC samples, tumors harboring the *BRAF* mutation showed low expression and poor membrane targeting of NIS in comparison with those without the mutation. This suggests that the *BRAF* mutation is an obstacle for membrane targeting 49. Additionally, treatment with a MEK inhibitor partially increased NIS expression, but did not allow NIS migration to the cell membrane in thyroid cells with the *BRAF* mutation.

A clinical study suggested that patients with significant expression of the proto-oncogene PTTG were closely associated with lymph node metastasis, distant metastasis, and advanced stages of thyroid cancer 211. Additionally, Heaney et al. reported PTTG overexpression in thyroid cancer tissue, which caused cellular transformation and dedifferentiation such as NIS repression and inhibition of RAI uptake 212. In thyroid cancer, PBF is upregulated and correlates with tumor growth, development, recurrence, and overall poorer disease outcome 28, 212, 213. There are two mechanisms for the repression of NIS expression and RAI uptake in overexpressed PBF. First, PBF overexpression represses NIS expression at the transcriptional level and reduces RAI uptake *in vitro* and *in vivo* (Figure 3D) 26, 214. Second, PBF overexpression causes the redistribution of NIS from the plasma membrane to the cytoplasm (Figure 4) 215. Smith et al. reported that binding and induction of Src kinase led to increased phosphorylation of PBF at residue Y174, triggering the repression of NIS retention in the plasma membrane and RAI uptake (Figure 4) 216. Abrogation of the Y174 residue by the Src kinase inhibitor (PP1) diminishes the interaction between PBF and NIS and increases RAI uptake.

Amit et al. reported the role of phosphatidylinositol glycan anchor biosynthesis class U (PIGU) expression for NIS trafficking to the cell membrane via the MAPK signaling pathway in PTC (Figure 4) 217. Glycosylphosphatidylinositol transamidase (GPIT) catalyzes the addition of glycosylphosphatidylinositol anchor to substrate proteins in the endoplasmic reticulum 218. A PIGU is a protein in the GPIT complex, and its expression was low in primary PTC and PTC cell lines compared with normal thyroid cells 217. The downregulation of PIGU inhibited NIS glycosylation and cell membrane targeting followed by impaired RAI uptake in PTC. Overexpression of PIGU by blockade of the MAPK signaling pathway restored NIS function. Moreover, PIGU-positive tumors in patients with tumor recurrence who were treated with RAI therapy correlated with positive RAI uptake and moderate-to-strong NIS expression and high Tg levels compared with patients with PIGU-negative tumors, suggesting it to be a novel predictor for response to RAI.

Lacoste et al. described sequestered NIS in the cytoplasm or impaired NIS movement to the cell membrane through an interaction between NIS and leukemia-associated RhoA guanine exchange factor (LARG) with sequestered NIS increasing tumor cell motility and invasion (Figure 4) 219. Recently, Feng et al. reported that *PTEN* alteration, a common somatic mutation, and upregulation of the PI3K/AKT signaling pathway likely inhibits NIS glycosylation which impairs its plasma membrane localization. This results in increased cytoplasmic NIS expression 220, 221. Additionally, *PTEN* alteration increases LARG expression and RhoA activation, resulting in enhanced cytoplasmic NIS expression through its interaction with NIS-LARG.

Fletcher et al. identified two novel substances that interact with NIS, ADP-ribosylation factor 4 (ARF4) and valosin-containing protein (VCP). Both have important roles in NIS movement to the plasma membrane and enhancement of RAI uptake (Figure 4) 222. ARF4 recognizes the VAPK motif in the NIS C-terminus and moves vesicular NIS to the plasma membrane. VCP, a component of ER-associated degradation, governs NIS proteolysis. The application of VCP inhibitors abrogated the inhibition of NIS function mediated by VCP and increased NIS expression at the plasma membrane followed by increased RAI uptake in thyroid cancer cells and primary thyrocytes isolated from a mouse model.

Huc-Brandt et al. suggested the dimerization of NIS based on electrophoresis patterns, size exclusion chromatography, and light scattering analyses 223. Additionally, Thompson et al. demonstrated the role of NIS dimerization for its movement to the plasma membrane, suggesting new mechanisms to be considered in treating patients with RAI-refractory thyroid cancer in the future 224.

## Clinical trials

Several clinical trials have been conducted that focus on dedifferentiation mechanisms to reinduce RAI avidity in progressive RAI-refractory thyroid cancers (Table 3).

Simon et al. administered 13-cis RA (isotretinoin) to patients with RAI-refractory thyroid cancer and found that RAI uptake was reinduced in 4 of 10 patients 225. Based on results of that trial, several additional studies also reported approximately 20%-40% improvement in RAI uptake after isotretinoin treatment 226-231. In addition, Handkiewicz-Junak et al. reported that increased RAI uptake was confirmed in 9 of 53 patients; however, no clinical benefit such as inducing tumor regression or preventing disease progression was found 232. Short et al. conducted an open-label, non-randomized, phase II trial with isotretinoin and found that RAI uptake increased in only 1 of 16 patients, showing a different response from that noted in other studies of isotretinoin application 233. Clinical trials with administration of other RAs such as RXR activator (bexarotene) and all-trans-RA (tretinoin) to patients with RAI-refractory thyroid cancer have also been reported by several groups 234-236. An open, prospective, intervention study reported that 8 of 11 patients with RAI-refractory DTC having metastases responded to bexarotene administration with restoration of RAI uptake 234. However, there was limited clinical relevance due to heterogenous responses of different metastases even in a patient with a low degree of RAI uptake. In 11 patients with advanced DTC, tretinoin treatment increased RAI uptake in 4 patients with a partial response of target lesions in 5 patients 235. In another study with RAI-refractory DTC patients, tretinoin treatment slightly increased RAI uptake in 6 patients, whereas 7 patients experienced no RAI uptake 236. A meta-analysis using 14 studies of RA treatment in RAI-refractory DTC revealed a pooled disease response rate assessed by RAI scintigraphy to be 27.6% 237. Moreover, the pooled response rate was only 17.0% using the Response Evaluation Criteria in Solid Tumours (RECIST) criteria and 30.8% using the World Health Organization criteria. These results suggest that only a few patients with RAI-refractory thyroid cancer respond to RA treatment.

The pilot study to demonstrate increased RAI uptake with rosiglitazone, a PPARɣ agonist which is a member of the thiazolidinedione class, was conducted by Philips et al., and it was found that only 1 of 5 patients had a slightly increased RAI uptake 238. Two case reports described the restoration of RAI uptake in RAI-refractory thyroid cancer following rosiglitazone treatment and decreased serum Tg and tumor size after RAI treatment 239, 240. In a phase II trial, Kebebew et al. reported that 4 of 10 patients with RAI-refractory thyroid cancer had positive RAI uptake following 7 weeks of rosiglitazone administration 241. In addition, another phase II trial by the same group reported that 5 of 20 patients had positive RAI uptake after rosiglitazone treatment 242. Tepmongkol et al. reported that 7 of 23 patients exhibited strongly positive staining for PPARɣ in thyroid biopsies after rosiglitazone administration 243. Among the 7 patients with positive PPARɣ staining, 5 had increased RAI uptake on RAI scan. Rosenbaum-Krumme et al. examined whether both rosiglitazone and pioglitazone have the ability to restore RAI uptake in patients with progressive RAI-refractory DTC 244, 245. They found that 5 of 9 patients exhibited improved RAI uptake. Of the 4 patients treated with RAI, 3 had a partial response when the RECIST 1.1 criteria were used 244. However, another study on pioglitazone treatment in 5 patients with progressive DTC found no increased RAI uptake 245.

Kelly et al. performed a phase I study with SAHA (vorinostat) as an HDAC inhibitor for advanced thyroid cancer patients and found that 1 of 3 patients with PTC exhibited increased RAI uptake after SAHA treatment 246. The results of romidepsin administration to patients with RAI-refractory thyroid cancer have also been reported 247, 248. In a phase I trial by Amiri-Kordestani et al., 2 of 6 patients with thyroid cancer exhibited slightly increased RAI uptake after romidepsin administration; however, the degree of RAI uptake was not sufficient to allow RAI therapy 247. In another phase II trial of romidepsin, 2 of 16 patients with RAI-refractory thyroid cancer had a reversal of RAI uptake; however, RAI treatment had no clinical benefit 248. Nilubol et al. tested the effect of restoring RAI avidity by valproic acid for patients with RAI-refractory thyroid cancer, and the results revealed that none of the 10 patients had increased RAI uptake 249.

Ho et al. reported promising outcomes with the administration of selumetinib as an MEK inhibitor to RAI-refractory DTC patients. They noted that 12 of 20 patients' recovered RAI uptake, and 8 of these 12 patients reached the dosimetry threshold for RAI therapy 7. Among these patients, 5 harbored the *NRAS* mutation and only 1 had the *BRAF* mutation, suggesting that the recovery of RAI avidity was higher in patients with *NRAS* mutations than in those with the *BRAF* mutation. Rothenberg et al. reported clinical trials on dabrafenib as a BRAF inhibitor to overcome RAI refractoriness in patients with *BRAF*-mutated thyroid cancer 250. Six weeks after the administration of dabrafenib to patients with *BRAF*-mutated thyroid cancer, 6 of 10 patients had restored RAI uptake, 2 exhibited a partial response, and 4 had stable disease. Dunn et al. published a pilot trial on utilizing vemurafenib for approximately 4 weeks in 12 patients with *BRAF*-mutated RAI-refractory PTC or PDTC 251. Of the 10 patients evaluated, 4 exhibited restored RAI uptake and RAI therapy was initiated, resulting in tumor regression. Moreover, the effect of vemurafenib to inhibit the MAPK signaling pathway was closely associated with thyroid differentiation score and RAI uptake. Jaber et al. analyzed the restoration of RAI uptake after the administration of BRAF and MEK inhibitors in 13 patients with RAI-refractory DTC or PDTC 252. Among these patients, 9 had the *BRAF* mutation, 3 had *RAS* mutations, and 1 had wild-type. Notably, 9 of 13 patients exhibited increased RAI uptake. In particular, the best responders were *RAS*-mutated patients (3 of 3 *RAS*-mutated patients, 5 of 9 *BRAF*-mutated patients, and 1 of 1 wild-type patients). Iravani et al. reviewed the restoration of RAI avidity by TKIs in patients with RAI-refractory DTC 10. MEK inhibitor alone was given to 3 patients with the *NRAS* mutation, and combined MEK and BRAF inhibitors were given to 3 patients with the *BRAF* mutation. This study found that 4 of 6 patients exhibited restoration of RAI avidity, and patients with the *BRAF* mutation exhibited a better response by restoring RAI avidity than patients with the *NRAS* mutation (3 of 3 vs. 1 of 3). Following RAI therapy, 3 patients exhibited a partial response and 1 patient had stable disease. Several clinical studies with multiple drugs that target the MAPK signaling pathway are currently ongoing (NCT02145143, NCT04462471, NCT03244956, NCT02152995 and ISRCTN17468602) (Table 4).

In addition to abovementioned clinical trials with regard to the MAPK signaling pathway, clinical trials targeting new therapeutic targets have also been conducted by several groups. Modoni et al. treated a patient with RAI-refractory PTC with nevirapine, a non-nucleoside reverse transcriptase inhibitor, and reported restoration of RAI uptake (Table 3) 253. Liu et al. reported that 7 of 12 patients with DTC exhibited increased RAI uptake after lithium treatment, but adding lithium to RAI therapy had no clinical benefits (Table 3) 254. Based on a preclinical study for regulating cell redifferentiation along with blockade of PDGFRɑ 110, a clinical trial is ongoing with the administration of imatinib as an anti-cancer drug by blocking PDGFRɑ function in patients with advanced thyroid cancer (NCT03469011) (Table 4).

## Future perspectives and conclusion

**Figure 5:**
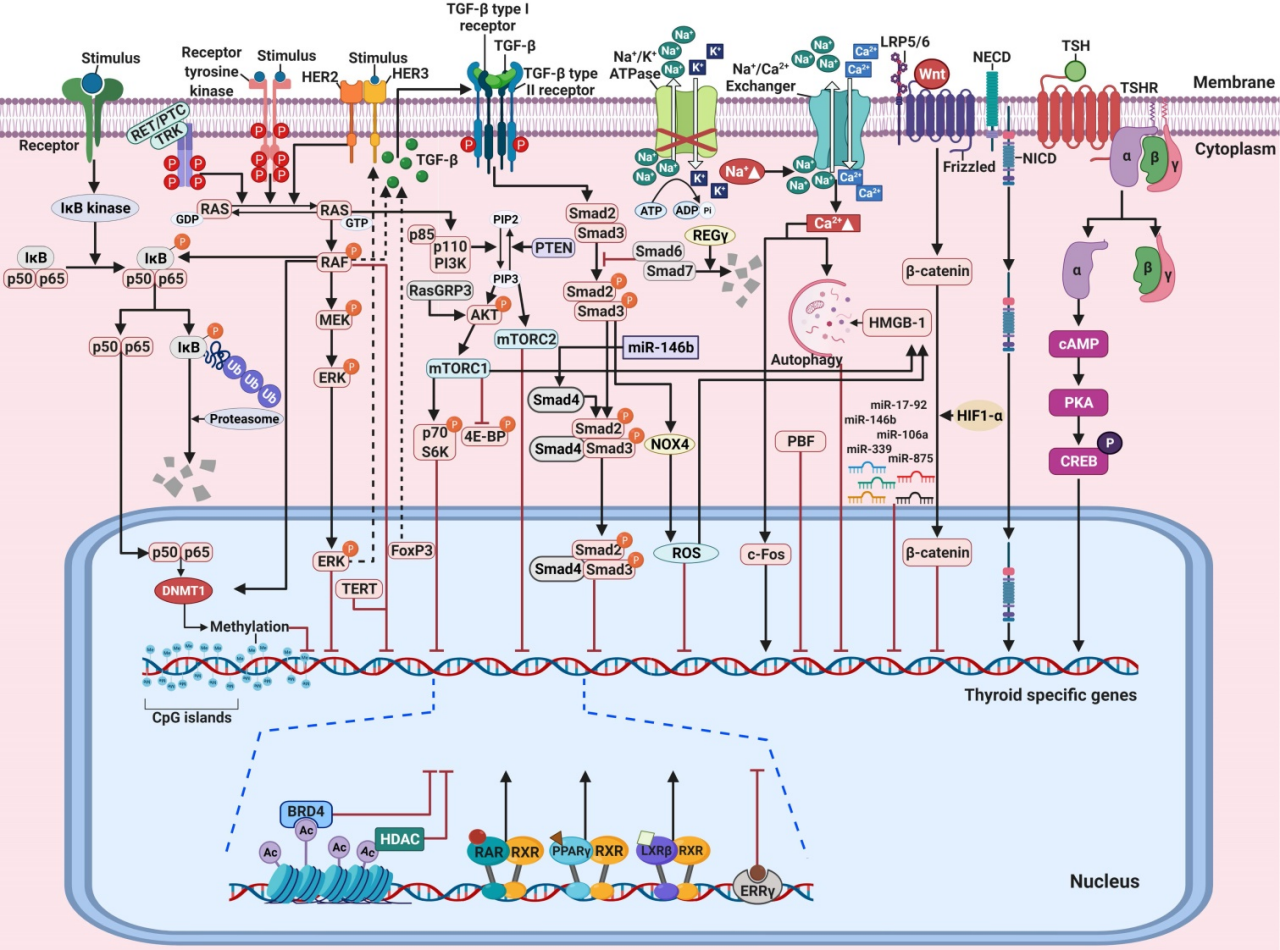
** Comprehensive molecular mechanisms related to RAI refractoriness in differentiated thyroid cancer.** Abbreviations: 4E-BP: 4E-binding protein; Ac: acetylation; BRD4: bromodomain-containing protein; DNMT1: DNA methyltransferase 1; ERRɣ: estrogen-related receptor ɣ; FoxP3: forkhead transcription factor 3; HDAC: histone deacetylase; HMGB1: high mobility group box 1; IκB: inhibitory κB; LXRß: liver X receptor ß; TERT: telomerase reverse transcriptase; TSHR: TSH receptor; miR: microRNA; mTORC1: mTOR complex 1; mTORC2: mTOR complex 2; P: phosphorylation. NECD: notch extracellular domain; NICD: notch intracellular domain; NOX4: NADPH oxidase 4; P: phosphorylation; PBF: pituitary tumor-transforming gene 1 (PTTG1)-binding factor; PTEN: phosphatase and tensin homolog; RAR: retinoic acid receptor; PPARɣ: peroxisome proliferator-activated receptor ɣ; ROS: reactive oxygen species; RAR: retinoic acid receptor; RXR: retinoid X receptor; TERT: telomerase reverse transcriptase; Ub: ubiquitination. Image was created with BioRender.com.

For a long time, RAI therapy has been successfully used as a treatment for thyroid cancer. It is administered after thyroidectomy for ablation of any remaining thyroid tissue and residual or metastatic cancer, and it has contributed to the excellent prognosis of DTCs. However, dedifferentiated thyroid cancers that lose RAI avidity are not responsive to RAI therapy and have a poor prognosis. Restoring RAI avidity via thyroid-specific gene expression is a promising strategy to convert RAI-refractory thyroid cancers into RAI-sensitive thyroid cancers. Thus, various compounds have been examined to restore RAI uptake for RAI non-avid thyroid cancers in both preclinical studies and clinical trials by multiple researchers. Unfortunately, there are no definitive successful clinical trials that meet the expectation of pharmacologic redifferentiation effects yet. There are several shortcomings and limitations of current studies regarding the development of innovative compounds for understanding the mechanisms underlying RAI-refractory thyroid cancer. First, most studies examined and focused on a specific signaling pathway but not comprehensive signaling pathways. However, RAI refractoriness is complex and closely associated with multiple signaling pathways. Therefore, sometimes, blockade of a single signaling pathway was insufficient to completely block a target. In addition, resistance or reactivation of specific signaling pathways after pharmacologic treatment could also be an obstacle for redifferentiation efficacy. As noted previously, treatment with a single drug could fail to meet the redifferentiation efficacy owing to reactivation of the signaling pathway followed by drug resistance 57, 63. Therefore, strategies with combination treatments to overcome resistance and decreased efficacy of drugs should be considered. Second, the TME can disturb the target drug response. Recently, an article reported that TME (hypoxia and quiescence) could influence NIS expression in exogenous *NIS*-expressing cancer cells. Specifically, immunohistochemistry with tumor tissue from an animal model showed heterogeneous characteristics within the peripheral region revealing stronger NIS expression with lower HIF-1ɑ levels than internal regions 210. In addition, most *in vitro* studies employed 2-dimensional monolayer culture with cancer cells. This environment may not reflect the heterogenous characteristics of cancer with a change in TME. Therefore, the development of experimental protocols using an environment similar to that of actual cancer tissue can be critical to the discovery of valuable drugs for RAI-refractory thyroid cancers. To address this concern, we should consider the following approaches for enhancing RAI uptake via redifferentiating agents in RAI-refractory thyroid cancers: (1) tailored redifferentiation strategy depending on genetic and epigenetic alteration because RAI-refractory thyroid cancers are quite heterogeneous genetically and epigenetically and their responses to redifferentiation therapy also are variable by their inborn nature; (2) combination strategy with >2 compounds for redifferentiation because most RAI-refractory thyroid cancers develop dedifferentiation by multiple factors; and (3) optimization of redifferentiation strategy for dosing and duration. Understanding the detailed molecular mechanisms for dedifferentiation of individual RAI-refractory thyroid cancers, such as genetic mutations, epigenetic alteration, change of signaling pathways, and posttranslational protein modification, is needed to develop the optimal personalized redifferentiation strategy (Figures 4 and 5). In the future, pharmacologic redifferentiation followed by RAI treatment can be one of the main therapeutic strategies for dedifferentiated RAI-refractory thyroid cancers.

## Figures and Tables

**Table 1:** Summary of preclinical studies with redifferentiation strategy for radioactive-iodine (RAI) refractoriness in differentiated thyroid cancer

Target	Compound	Type of study	Result	Reference
Autophagy	Digitalis-like compounds (Digoxin, Digoxigenin, Proscillaridin A, Lanatoside C, Strophantin K)	*In vitro*	*TTF-1, TTF-2, PAX-8, Tg* ↑ NIS ↑ ^125^I uptake ↑	182, 183
BRD4	JQ1	*In vitro, In vivo*	NIS ↑ ^131^I uptake ↑	186
BRAF	PLX4720	*In vivo*	*NIS*, *TPO*, *Tg*, *TSHR*, *PAX-8*, *TTF-1* ↑ NIS ↑ ^124^I uptake ↑ ^131^I cytotoxicity ↑	58
DNA methylation	5-aza-2'-deoxycytidine	*In vitro*	*NIS* ↑ ^125^I uptake ↑	132
	5-azacytidine	*In vitro*	*NIS* ↑ ^125^I uptake ↑	135
ERRɣ	Compound 35	*In vitro, In vivo*	NIS, TPO, TSHR, Tg ↑ ^124^I, ^125^I uptake ↑ ^131^I cytotoxicity ↑	177
	DN200434	*In vitro, In vivo*	NIS, TPO, TSHR, Tg ↑ ^124^I, ^125^I uptake ↑ ^131^I cytotoxicity ↑	176
	GSK5182	*In vitro*	NIS (membrane) ↑ ^125^I uptake ↑ ^131^I cytotoxicity ↑	175
HDAC	AB2, AB3 and AB10	*In vitro*	*NIS*, *PAX-8*, *TTF-1*, *TTF-2, TSHR* ↑	152
	APHA compound 8	*In vitro*	NIS ↑ ^125^I uptake ↑	148
	Apicidine	*In vitro*	NIS ↑ ^125^I uptake ↑	148
	Depsipeptide	*In vitro, In vivo*	NIS, Tg, TPO ↑ ^125^I uptake ↑	143, 144
	Entinostat	*In vitro*	^125^I uptake ↑	149
	Panobinostat (LBH589)	*In vitro*	NIS ↑ ^125^I uptake ↑ ^131^I cytotoxicity ↑	145
	Sodium butyrate	*In vitro*	*NIS*,* PAX-8* ↑	150
	Trichostatin A	*In vitro*	*NIS* ↑ NIS, Tg, TPO ↑	146, 150, 151
	Valproic acid	*In vitro*	*Tg* ↑ NIS ↑ ^125^I uptake ↑	144, 147, 148
	Vorinostat	*In vitro*	*NIS*,* TSHR, TPO* ↑	16
LXR	Dendrogenin A	*In vitro*	NIS, TPO, TSHR, Tg ↑ ^123^I, ^131^I uptake ↑	173
MAPK	CTOM-DHP	*In vitro, In vivo*	NIS, TPO, Tg, TSHR, PAX-8, TTF-1 ↑ ^123^I, ^125^I uptake ↑ ^131^I cytotoxicity ↑ Tumor growth ↓	30
	K905-0266	*In vitro, In vivo*	NIS, TPO, Tg, TSHR, PAX-8, TTF-1 ↑ ^125^I uptake ↑ ^131^I cytotoxicity ↑ Tumor burden ↓	84
MAPK, SAPK/JNK	Sunitinib	*In vitro*	*NIS*, *TPO*, *Tg*, *TSHR*, *PAX-8*↑	81
MEK	CH5126766	*In vivo*	NIS ↑ ^124^I uptake ↑ ^131^I cytotoxicity ↑ Tumor growth ↓	83
	PD0325901	*In vivo*	*NIS*, *TPO*, *Tg*, *TSHR*, *PAX-8*, *TTF-1* ↑ NIS ↑ ^124^I uptake ↑ ^131^I cytotoxicity ↑	58
	Selumetinib	*In vivo*	NIS ↑ ^124^I uptake ↑ ^131^I cytotoxicity ↑ Tumor growth ↓	83
	U0126	*In vitro*	*TSHR*, *NIS*, *Tg* ↑	56
mTORC1	Rapamycin	*In vitro*	NIS ↑ *TTF-1*, *TTF-2*, *PAX-8*, *TSHR* ↑ ^125^I uptake ↑	92
mTORC1/mTORC2	Torin2	*In vitro*	*NIS* ↑	94
Notch	Resveratrol	*In vitro*	*NIS*,* TTF-1*, *TTF-2*, *PAX-8* ↑	96
PI3K	LY294002	*In vitro*	NIS ↑ ^125^I uptake ↑	89
PPARɣ	Pioglitazone, Rosiglitazone	*In vitro*	^125^I uptake ↑	168, 169
	Rosiglitazone	*In vitro*	^125^I uptake ↑	148
	Troglitazone	*In vitro*	NIS, Tg ↑ ^125^I uptake ↑	168, 169
Retinoic acid receptor	13-cis retinoic acid	*In vitro*	^131^I uptake ↑	163
	All-trans retinoic acid	*In vitro*	*NIS, Tg* ↑ NIS ↑ ^125^I uptake ↑	148, 164-166
	All-trans retinol	*In vitro*	*NIS* ↑ ^125^I uptake ↑	148
Reverse transcriptase enzyme	Nevirapine	*In vitro, In vivo*	NIS, PAX-8, TSHR ↑ ^125^I uptake ↑	17, 201
VEGFR, RET, MET, FLT3, AXL	Cabozantinib	*In vitro*	NIS, TPO, Tg, TSHR ↑ ^125^I uptake ↑ ^18^F-FDG uptake ↓	80
VEGFR, PDGFR, RET, KIT, FLT	Sorafenib	*In vitro*	NIS, TPO, Tg, TSHR ↑ ^125^I uptake ↑ ^18^F-FDG uptake ↓	80

Abbreviations: BRD4: bromodomain-containing protein 4; ERRɣ: estrogen-related receptor ɣ; FLT: fms-related receptor tyrosine kinase; ^18^F-FDG: fluorodeoxyglucose (^18^F); FLT-3: fms-related receptor tyrosine kinase-3; HDAC: histone deacetylase; LXR: liver X receptor; MAPK: mitogen-activated protein kinase; MET: MNNG HOS transforming gene; NIS: sodium iodide symporter; PAX-8: paired box gene-8; PDGFR: platelet-derived growth factor receptor; PI3K: phosphoinositide 3-kinase; PPARɣ: peroxisome proliferator-activated receptor ɣ; Tg: thyroglobulin; TPO: thyroperoxidase; TSHR: thyroid-stimulating hormone (TSH) receptor; TTF-1: thyroid transcription factor-1; TTF-2: thyroid transcription factor-2; VEGFR: vascular endothelial growth factor receptor.

**Table 2:** Summary of preclinical studies with combination treatment for redifferentiation of radioactive-iodine (RAI) refractoriness in differentiated thyroid cancer

Target	Compound	Type of study	Result	Reference
BRAF, HER, MEK	Dabrafenib, Lapatinib, Selumetinib, Lapatinib	*In vitro*	NIS, TPO, Tg, TSHR ↑ ^125^I uptake ↑ ^131^I cytotoxicity ↑	57
BRAF, MEK	Vemurafenib, PD98059	*In vitro*	NIS ↑ ^125^I uptake ↑	63
DNA methylation, HDAC	5-aza-2'-deoxycytidine, Sodium butyrate	*In vitro*	*NIS* ↑ ^125^I uptake ↑	156
	5-aza-2'-deoxycytidine, Valproic acid	*In vitro*	*NIS* ↑ ^125^I uptake ↑	157
HDAC, MAPK	Vemurafenib, Vorinostat	*In vitro*	NIS, TPO, Tg, TSHR ↑ ^125^I uptake ↑	19
	Selumetinib, Dabrafenib, Panobinostat	*In vitro*	*NIS*,* Tg, TSHR, TPO* ↑ NIS ↑ ^125^I uptake ↑ ^131^I cytotoxicity ↑	154
HDAC, MAPK, PI3K/AKT	RDEA119, Temsirolimus, Perifosine, Vorinostat	*In vitro*	*NIS*,* TSHR, TPO* ↑ NIS ↑ ^125^I uptake ↑	16
HDAC, Retinoic acid receptor	Tributyrin, Retinoic acid	*In vitro*	NIS ↑ ^125^I uptake ↑	155
HMT, MAPK	Dabrafenib, Selumetinib, Tazemetostat	*In vitro*	NIS, TPO, Tg, TSHR, PAX-8, TTF-1 ↑ ^125^I uptake ↑ ^131^I therapy ↑	158
MAPK, SAPK/JNK	Sunitinib, Forskolin	*In vitro*	*NIS*, *TPO*, *Tg*, *TSHR*, *PAX-8* ↑	81

Abbreviations: HDAC: histone deacetylase; HER: human epidermal growth factor receptor; HMT: histone methyltransferase; MAPK: mitogen-activated protein kinase; NIS: sodium iodide symporter; PAX-8: paired box gene-8; PI3K: phosphoinositide 3-kinase; SAPK/JNK: stress-activated protein kinases/jun amino-terminal kinases; Tg: thyroglobulin; TPO: thyroperoxidase; TSHR: thyroid-stimulating hormone (TSH) receptor; TTF-1: thyroid transcription factor-1.

**Table 3:** Update of clinical trials of redifferentiation strategy for radioactive-iodine (RAI) refractory differentiated thyroid cancers

Classification	Drug	Type of study	Patients (n)	Dose	Duration of treatment before RAI	Main Result	Reference
BRAF inhibitor	Dabrafenib	Interventional study	10	150 mg	25 days	Increase of RAI uptake (6/10); PR (2/6) and SD (4/6) after RAI therapy	250
	Vemurafenib	Pilot study	Total 12 10 evaluable	960 mg	4 weeks	Increase of RAI uptake (4/10); Tumor regression after RAI therapy (3/4)	251
HDAC inhibitor	Romidepsin (Depsipeptide)	Phase I	11	1 to 9 mg/m^2^	6-112 weeks (21 days cycle)	Faintly increase of RAI uptake (2/6)	247
		Phase II	20	13 mg/m^2^	0.46-12 months (28 days cycle)	Increase of RAI uptake (2/16)	248
	Valproic acid	Phase II	13	500 mg once for 3 days followed by 500 mg	10 weeks	Increase of RAI uptake (0/10)	249
	Vorinostat	Phase I	6	200-400 mg	12-37+ months (4 weeks cycle)	Increase of RAI uptake (1/3)	246
-	Lithium	Pilot study	12	1200 mg	Unknown duration (During and after 2^nd^ RAI therapy)	Increase of RAI uptake (5/12); ^*^Mixed pattern of RAI uptake (2/12); Response for RAI therapy (0/12).	254
MEK inhibitor	Selumetinib	Pilot study	Total 24 20 evaluable	75 mg	4 weeks	Increase of RAI uptake (12/20); Reached dosimetry threshold for RAI therapy (8/12); PR (5/8) and SD (3/8) after RAI therapy.	7
Non-nucleoside reverse transcriptase inhibitor	Nevirapine	Case report	1	200 mg	Unknown duration	Increase of RAI uptake in metastatic sites	253
PPARɣ agonist	Pioglitazone	Case report	5	30 mg followed by 45 mg	6 months (30 mg dose for 2 weeks followed by 45 mg dose)	Increase of RAI uptake (0/5)	245
	Rosiglitazone	Case report	1	8 mg	3 months	Increase of RAI uptake	239
		Case report	1	8 mg	2 months	Increase of RAI uptake; Tumor regression after RAI therapy.	240
		Phase II	10	4 mg or 8 mg	1 week for 4 mg dose; 7 weeks for 8 mg dose	Increase of RAI uptake (4/10)	241
		Phase II	20	4 mg or 8 mg	1 week for 4 mg dose; 7 weeks for 8 mg dose	PR for RAI uptake and Tg level (5/20)	242
PPARɣ agonist	Rosiglitazone	Pilot study	5	4 mg or 8 mg	1 month for 4 mg dose; 2 months for 8 mg dose	Faintly increase of RAI uptake (1/5)	238
		Pilot study	23	8 mg	6 weeks	Increase of RAI uptake (5/23)	243
		Pilot study	9	4 mg followed by 8 mg	6 months (4 mg dose for 2 weeks followed by 8 mg dose)	Increase of RAI uptake (5/9); Tumor regression after RAI therapy (3/9)	244
Retinoic acids	Bexarotene	Pilot study	12	300 mg	6 weeks	PR for RAI uptake (8/11)	234
	Isotretinoin	Pilot study	10	1.5 mg/kg	6 weeks	Increase of RAI uptake (4/10)	225
		Pilot study	50	1.5 mg/kg	5 weeks	Increase of RAI uptake (21/50); Tumor regression after RAI therapy (6/37)	226
		Pilot study	25	1 mg/kg	3 months	Increase of RAI uptake (5/25)	227
		Pilot study	5	1.0 to 1.5 mg/kg	5 weeks	Increase of RAI uptake (3/5); Decrease of tumor size (1/5)	228
		Pilot study	11	1.5 mg/kg	8 weeks	Increase of RAI uptake (2/11)	229
		Pilot study	27	0.66-1.5 mg/kg	5-12 weeks	Optimal positive RAI uptake (9/27); Suboptimal RAI uptake (10/27); Tumor regression or stabilization after RAI therapy (7/17)	230
		Pilot study	47	1-1.5 mg/kg	6 weeks	CR for RAI uptake (1/47); PR for RAI uptake (9/47)	231
		Phase II	53	1.0 mg/kg/day for 1 week followed by 1.5 mg/kg	6 weeks	Increase of RAI uptake (9/53)	232
		Phase II	16	1.5 mg/kg	8 weeks	Increase of RAI uptake (1/16)	233
	Tretinoin	Pilot study	13	1.5 mg/kg	1.5-18 months	Faintly increase of RAI uptake (6/13); PR for RAI therapy (1/13)	236
		Retrospective study	11	1.00±0.09 mg·kg^-1^·d^-1^	30 or 60 days	Re-induction of RAI uptake (4/11); PR for RAI therapy (5/11)	235
Combination	Dabrafenib ± Trametinib, Vemurafenib, Trametinib, Investigational MEK inhibitor	Retrospective study	13	Unknown	Median duration of treatment: 14.3 months (range, 0.9 to 76.4)	Increase of RAI uptake (9/13); PR (3/9) and SD (6/9) after RAI therapy	252
	Trametinib ± Dabrafenib, Vemurafenib + Cobimetinib	Retrospective, cohort study	6	Unknown	4 weeks	Increase of RAI uptake (4/6); PR (3/4) and SD (1/4) after RAI therapy	10

Abbreviations: CR: complete response; HDAC: histone deacetylase; N/A: not available; PPARɣ: peroxisome proliferator-activated receptor; PR: partial response; RAI: radioactive iodine; SD: stable disease. *Mixed pattern means 2 regions of interest with different effects of lithium.

**Table 4:** Currently ongoing clinical trials of redifferentiation strategy for radioiodine refractoriness in differentiated thyroid cancer

Identifier	Target	Drug	Type of Study	Enrolled Patients	Primary Outcome	Status
NCT02145143	BRAF	Vemurafenib	Pilot study, Single arm, Open label	12 (*BRAF* mutant, RAI-refractory thyroid cancers)	*Duration of overall response	Estimated study completion date: May, 2021
NCT04462471	BRAF, PI3K	Vemurafenib+ Copanlisib	Phase I, Single group assignment, Open label	22 (*BRAF* mutant, RAI-refractory thyroid cancers)	MTD	Estimated study completion date: June, 2022
NCT03244956	MEK, BRAF	Trametinib or Dabrafenib	Phase II, Parallel assignment, Open label	87 (one for patients with *RAS* mutation and one for patients with *BRAF^V600E^* mutation)	ORR	Estimated study completion date: December, 2022
NCT02152995	MEK	Trametinib	Phase II, Single arm, Open label	35 (*RAS* mutant or *RAS/RAF* wild-type, RAI-refractory recurrent and/or metastatic thyroid cancers)	Proportion of patients alive following treatment with trametinib and 124I, PFS, ORR, Iodine incorporation	Estimated primary completion date: June 30, 2021
ISRCTN17468602	MEK	Selumetinib	Phase II, Single arm,	80 (Differentiated thyroid cancer)	PFS	Closed (Overall trial end date: 18/01/2020)
NCT03469011	PDGFRα	Imatinib	Phase I, Sequential assignment, Open label	18 (Metastatic thyroid cancer)	Restore iodine uptake	Estimated study completion date: December 31, 2020

Abbreviations: MTD: maximum tolerated dose; ORR: objective response rate; PDGFRɑ: platelet-derived growth factor receptor-ɑ; PFS: progression-free survival; PI3K: phosphoinositide 3-kinase; RAI: radioactive iodine. * The duration of overall response is measured from the time measurement criteria are met for complete response or partial response (whichever is first recorded) until the first date that recurrent or progressive disease is objectively documented.

## Abbreviations

4E-BP1: 4E-binding protein 1
ARF4: ADP-ribosylation factor 4
ATC: anaplastic thyroid cancer
BRD4: bromodomain-containing protein 4
cAMP: cyclic adenosine monophosphate
CREB: cAMP response element-binding protein
DEHAL1: iodotyrosine dehalogenase 1
DIO2: iodothyronine deiodinase 2
DLC: digitalis-like compound
DNMT1: DNA methyltransferase 1
DTC: differentiated thyroid cancers
ERRs: estrogen-related receptors
ERKs: extracellular-signal-regulated kinases
FDG: fluorodeoxyglucose
FoxP3: Forkhead transcription factor 3
FTC: follicular thyroid cancer
GPIT: glycosylphosphatidylinositol transamidase
H3K18ac: H3 histone
H3K9-K14ac: acetylated lysines 9 and 14 in H3
HDAC: histone deacetylase
HER3: human epidermal growth factor receptor 3
HMGB1: high mobility group box 1
IκB: inhibitory κB
IKK: IκB kinase
JNK: Jun amino-terminal kinase
LARG: leukemia-associated RhoA guanine exchange factor
LPS: lipopolysaccharide
LXRs: liver X receptors
MAPK: mitogen-activated protein kinase
miRNA: microRNA
mRNA: messenger RNA
MTC: medullary thyroid cancer
mTOR: mechanistic target of rapamycin
mTORC1: mTOR complex 1
mTORC2: mTOR complex 2
NECD: notch extracellular domain
NF-κB: nuclear factor kappa-light-chain-enhancer of activated B cells
NICD3: notch intracellular domain 3
NIS: sodium iodide symporter
NOX4: NADPH oxidase 4
NUE: NIS upstream enhancer
SAPK: stress-activated protein kinase
PAX-8: paired box gene-8
PBF: PTTG1-binding factor
PDGFRɑ: platelet-derived growth factor receptor-ɑ
PDTC: poorly differentiated thyroid cancer
PI3K: phosphoinositide 3-kinase
PIGU: phosphatidylinositol glycan anchor biosynthesis class U
PKA: protein kinase A
PPARɣ: PAX8-peroxisome proliferator-activated receptor ɣ
PTC: papillary thyroid cancer
PTTG1: pituitary tumor-transforming gene 1
RA: retinoic acid
RAI: radioactive iodine
RAR: RA receptor
RECIST: response evaluation criteria in solid tumors
Ref-1: redox effector factor-1
ROS: reactive oxygen species
RXR: retinoid X receptor
TERT: telomerase reverse transcriptase
Tg: thyroglobulin
TGF-ß: transforming growth factor-ß
TLR4: toll-like receptor 4
TME: tumor microenvironment
TNF-ɑ: tumor necrosis factor-ɑ
TPO: thyroperoxidase
TSH: thyroid-stimulating hormone
TSHR: TSH receptor
TTF-1: thyroid transcription factor-1
TTF-2: thyroid transcription factor-2
VCP: valosin-containing protein
